# Solid–State Hydrogen Storage Materials with Excellent Selective Hydrogen Adsorption in the Presence of Alkanes, Oxygen, and Carbon Dioxide by Atomic Layer Amorphous Al_2_O_3_ Encapsulation

**DOI:** 10.1007/s40820-025-01934-7

**Published:** 2025-10-24

**Authors:** Fanqi Bu, Zhenyu Wang, Ali Wajid, Rui Zhai, Ting Liu, Yaohua Li, Xin Ji, Xin Liu, Shujiang Ding, Yonghong Cheng, Jinying Zhang

**Affiliations:** 1 State Key Laboratory of Electrical Insulation and Power Equipment, Center of Nanomaterials for Renewable Energy (CNRE), School of Electrical Engineering, Xi’an Jiaotong University, Xi’an, Shaanxi 710049 People’s Republic of China; 2 School of Chemistry, Xi’an Key Laboratory of Sustainable Energy Materials Chemistry, State Key Laboratory for Mechanical Behavior of Materials, Xi’an Jiaotong University, Xi’an, 710049 People’s Republic of China; 3 Aviation Engineering School, Air Force Engineering University, Xi’an, 710038 People’s Republic of China

**Keywords:** Hydrogen storage, Magnesium hydrides, Selective hydrogen adsorption, Air stability, Amorphous Al_2_O_3_ shells

## Abstract

Metal hydrides with high hydrogen density provide promising hydrogen storage paths for hydrogen transportation. However, the requirement of highly pure H_2_ for re-hydrogenation limits its wide application. Here, amorphous Al_2_O_3_ shells (10 nm) were deposited on the surface of highly active hydrogen storage material particles (MgH_2_–ZrTi) by atomic layer deposition to obtain MgH_2_–ZrTi@Al_2_O_3_, which have been demonstrated to be air stable with selective adsorption of H_2_ under a hydrogen atmosphere with different impurities (CH_4_, O_2_, N_2_, and CO_2_). About 4.79 wt% H_2_ was adsorbed by MgH_2_–ZrTi@10nmAl_2_O_3_ at 75 °C under 10%CH_4_ + 90%H_2_ atmosphere within 3 h with no kinetic or density decay after 5 cycles (~ 100% capacity retention). Furthermore, about 4 wt% of H_2_ was absorbed by MgH_2_–ZrTi@10nmAl_2_O_3_ under 0.1%O_2_ + 0.4%N_2_ + 99.5%H_2_ and 0.1%CO_2_ + 0.4%N_2_ + 99.5%H_2_ atmospheres at 100 °C within 0.5 h, respectively, demonstrating the selective hydrogen absorption of MgH_2_–ZrTi@10nmAl_2_O_3_ in both oxygen-containing and carbon dioxide-containing atmospheres hydrogen atmosphere. The absorption and desorption curves of MgH_2_–ZrTi@10nmAl_2_O_3_ with and without absorption in pure hydrogen and then in 21%O_2_ + 79%N_2_ for 1 h were found to overlap, further confirming the successful shielding effect of Al_2_O_3_ shells against O_2_ and N_2_. The MgH_2_–ZrTi@10nmAl_2_O_3_ has been demonstrated to be air stable and have excellent selective hydrogen absorption performance under the atmosphere with CH_4_, O_2_, N_2_, and CO_2_.

**Supplementary Information:**

The online version contains supplementary material available at 10.1007/s40820-025-01934-7.

## Highlights

- Gas selective amorphous Al_2_O_3_ encapsulation was constructed on highly reactive MgH_2_ using atomic layer deposition.
- Hydrogen selective adsorption was achieved in the impure hydrogen atmosphere containing impurities (O_2_, N_2_, CH_4_, and CO_2_).
- Excellent air stability with no MgO or Mg(OH)_2_ generated after 3 months of air exposure was achieved.

**Supplementary Information:**

The online version contains supplementary material available at 10.1007/s40820-025-01934-7.

## Graphical abstract

**Figure:**
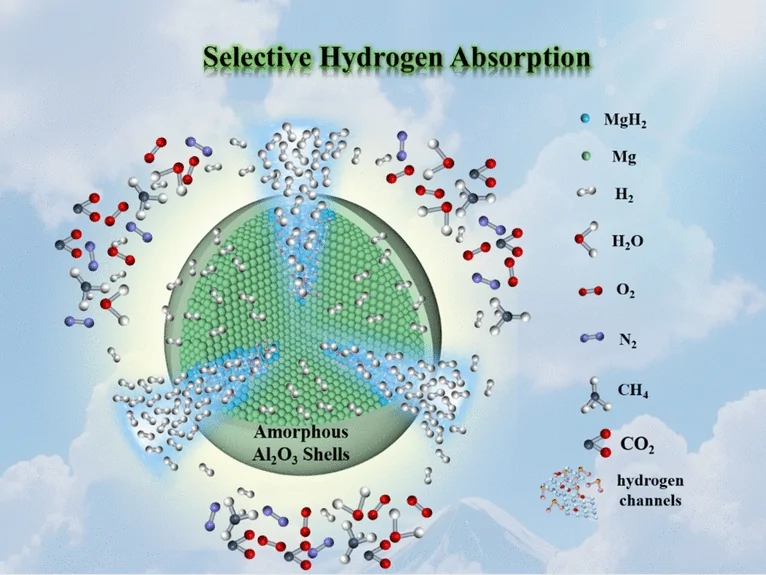

## Introduction

The H_2_ is expected to play an important role in the future energy system as a clean renewable energy carrier with high energy density [1]. However, the storage and transportation of H_2_ is still a bottleneck for the application of hydrogen energy. Metal hydrides with high hydrogen density are crucial for hydrogen storage and transportation. However, hydrogenation of metal hydrides with high hydrogen density must be conducted in a pure H_2_ atmosphere (99.999%) to prevent poisoning [2, 3]. The MgH_2_ is a typical metal hydride with high hydrogen storage density, which was demonstrated to provide a high theoretical gravimetric (∼7.6 wt% H_2_) as well as volumetric (∼110 kg m^−3^ H_2_) hydrogen storage density. However, the application of MgH_2_ is still limited to high dehydrogenation temperature, slow kinetics, and degrading cycling performance, especially the requirement of high pure H_2_ for hydrogenation due to its high reactivity. Various modifications including alloying [4, 5], nanosizing [6–9], and catalysts [2, 3, 10, 11] have been tried to enhance the kinetic properties of MgH_2_. The catalysts, especially transition metal-based catalysts (Ti, Zr, etc.) with 3*d* or 4*d* electronic orbitals, have been demonstrated to significantly enhance the kinetic of MgH_2_ [2, 10, 12–14]. Highly efficient reversible hydrogen storage in magnesium-based composites has been achieved through solar radiation under effective catalyst modification [15–17]. However, the stability of MgH_2_/Mg under different atmospheres has only been reported in very few studies in the form of air stability [18–20]. Nevertheless, the selective hydrogen absorption by hydrogen storage materials with high storage density in complex hydrogen atmospheres has not yet been realized and rarely been attempted.

The polymer encapsulation [19] and carbon layer coating [18, 20] were attempted to solve the air sensitivity of hydrogen storage materials. The Magnesium nanoparticles (~ 5 nm) were synthesized in poly(methyl methacrylate) (PMMA) matrix to enhance their air stability by a liquid-phase reaction method to release 4.64 wt% H_2_ [19], where a small amount of MgO and Mg(OH)_2_ were still detected after air exposure for 3 days. The Mg nanoparticles were also tried to be coated by carbon shells by a methane plasma metal reaction method to achieve extremely high air stability [18], where no MgO was detected after being exposed to air for 3 months. However, the dehydrogenation temperature and kinetic were found to be unsatisfactory, with 5 wt% of H_2_ released from Mg@C at 350 °C for 20 min. The MgH_2_ particles were also encapsulated in graphene nanorods by a wet chemical reduction method to achieve high air-stability [20], while about 5.3 wt% of H_2_ was observed to be released at 300 °C for 45 min. Bulk magnesium-nickel-based hydride through water treatment to enhance its air stability [5], while a hydrogen storage density of 3.5 wt% was obtained due to the weight of Ni [5, 21]. The air stability of Mg/MgH_2_ has been demonstrated to be enhanced to some extent with sacrifice of dehydrogenation kinetic and density. Various transition metal-based catalysts (such as Zr and Ti) have been demonstrated to effectively improve the dehydrogenation kinetics of hydrogen storage materials [2, 3, 6, 9, 21], which could be adopted to enhance the hydrogen storage performance of MgH_2_.

Most importantly, the hydrogenation of magnesium-based composites must be performed under a pure H_2_ atmosphere to prevent the poisoning of hydrogen storage materials [2, 3, 7, 8, 22]. The selective storage of hydrogen in a complex atmosphere is extremely important for the wide application of hydrogen storage materials, which could even be directly applied to industrial by-product hydrogen [23]. Alkanes are one of the main impurities in by-product hydrogen, where 24–28% CH_4_ is included in coke oven gas [24]. However, the CH_4_ was found to react with MgH_2_ even under 1.0 bar CH_4_ to significantly reduce the hydrogen storage performance of MgH_2_ [25]. The other impurities (such as oxygen or water) are more reactive with MgH_2_ [19, 20], which was usually avoided for hydrogen storage materials.

Here, we report a method to encapsulate MgH_2_ particles with homogeneously distributed hydrogen channels (MgH_2_–ZrTi) by atomic layers of amorphous Al_2_O_3_ shells through atomic layer deposition (ALD) to obtain MgH_2_–ZrTi@Al_2_O_3_. The amorphous Al_2_O_3_ shells were found to be inert, effectively shielding against H_2_O, O_2_, N_2_, CH_4_, and CO_2_, while allowing H_2_ to penetrate easily. The selective hydrogen absorption of MgH_2_–ZrTi@10nmAl_2_O_3_ has been demonstrated under hydrogen atmospheres with 10% CH_4_, 0.1% CO_2_, and varying contents of O_2_ and N_2_.

## Experimental Details

### Preparation of MgH_2_–ZrTi@Al_2_O_3_ and Mg–ZrTi@Al_2_O_3_

The commercial MgH_2_ (MG power Corp) was first coated with 10 nm ZrO_2_ by ALD, ball milled with few layers Ti_3_C_2_ (FL–Ti_3_C_2_) with the weight ratio of 19:1 to obtain MgH_2_–ZrTi according to our reported work [26]. The as-produced MgH_2_–ZrTi was then coated by amorphous Al_2_O_3_ by ALD. Specifically, 0.25 g of MgH_2_–ZrTi powder was placed in a glass vial containing 10 mL of anhydrous cyclohexane in the glove box and sealed using parafilm. The glass vial containing the mixture was sonicated for 30 min under ice bath, then subsequently added dropwise to a silicon wafer which was heated to 100 °C in the glove box. After the cyclohexane on the wafers was completely evaporated, the wafers with MgH_2_–ZrTi attached were placed in custom-made steel containers and quickly transferred to the deposition chamber of the ALD system. The ALD process was carried out cyclically, with an average Al_2_O_3_ deposition thickness of 0.1 nm for a single ALD cycle. The single ALD deposition process for Al_2_O_3_ was as follows: An trimethylaluminum (TMA) pulse for 30 ms, followed by an N_2_ purge for 1.97 s. And an oxygen plasma pulse for 5 s followed by an N_2_ purge for 1 s. The resulting samples were named MgH_2_–ZrTi@5nmAl_2_O_3_, MgH_2_–ZrTi@10nmAl_2_O_3_, and MgH_2_–ZrTi@20nmAl_2_O_3_, respectively, based on the deposited thickness of Al_2_O_3_. The MgH_2_–ZrTi after dehydrogenation at 300 °C was coated with 10 nm Al_2_O_3_ by ALD to prepare Mg–ZrTi@10nmAl_2_O_3_ in non-hydrogen state.

### Air Stability Tests of MgH_2_–ZrTi@Al_2_O_3_

The MgH_2_–ZrTi@Al_2_O_3_ samples obtained from ALD deposition were directly exposed to air, and the exposure times were set to 1 day, 1 week, 1 month, 2 months, and 3 months, respectively. The average temperature was 15 °C and the average humidity was 25% RH.

### Hydrogen Selective Adsorption Tests of MgH_2_–ZrTi@Al_2_O_3_

Hydrogen selective adsorption tests were performed in 10%CH_4_ + 90%H_2_ and 0.1%O_2_ + 0.4%N_2_ + 99.5%H_2_ atmosphere at different temperatures. The 0.1%O_2_ + 0.4%N_2_ + 99.5%H_2_ mixture was obtained by injecting H_2_ into a vessel containing 0.1 bar of standard air (21%O_2_ + 79%N_2_) until the pressure in the vessel reached 21 bar. The 0.1%CO_2_ + 0.4%N_2_ + 99.5%H_2_ mixture was obtained by injecting H_2_ into a vessel containing 0.1 bar of 21%CO_2_ + 79%N_2_ until the pressure in the vessel reached 21 bar. The high concentration of O_2_ was performed using a two-step method for hydrogenation, where the sample was first heated under pure hydrogen and followed by 21%O_2_ + 79%N_2_ at 75 °C for 1 h.

## Results and Discussion

### Structure Characterization of MgH_2_–ZrTi@Al_2_O_3_

**Fig. 1:**
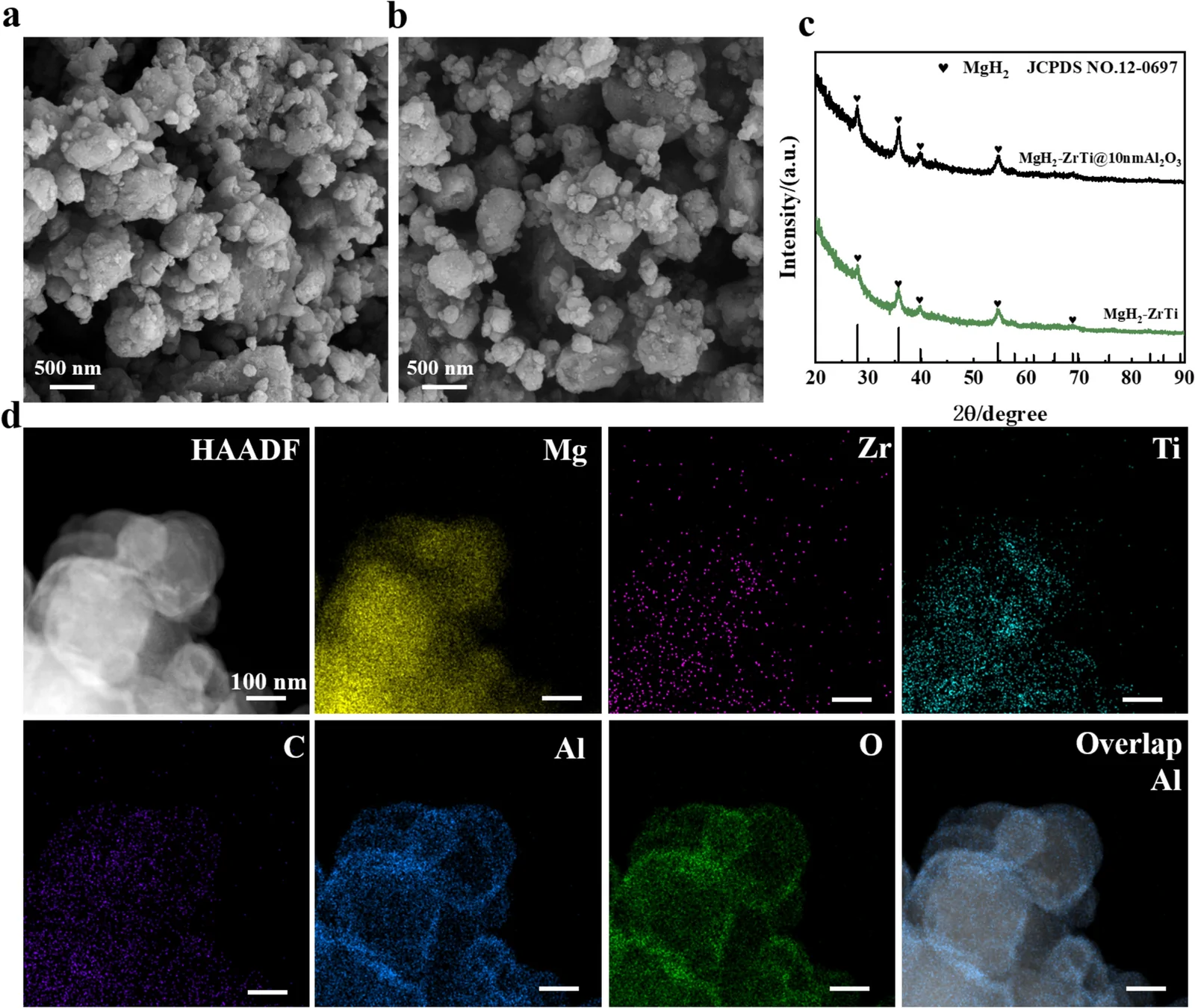
Characterization of MgH_2_–ZrTi and MgH_2_–ZrTi@10nmAl_2_O_3_. SEM image of **a** MgH_2_–ZrTi and **b** MgH_2_–ZrTi@10nmAl_2_O_3_. **c** XRD spectra of MgH_2_–ZrTi (green) and MgH_2_–ZrTi@10nmAl_2_O_3_ (black). **d** HAADF-STEM and elemental mapping analysis of MgH_2_–ZrTi@10nmAl_2_O_3_

The MgH_2_–ZrTi with low reaction temperature, fast kinetics as well as stable cycling properties were prepared by introducing oxygen-rich vacancies of Zr, Ti components to MgH_2_ in our previous study [26]. Amorphous Al_2_O_3_ shells were constructed on MgH_2_–ZrTi particles by ALD to achieve selective hydrogen absorption at moderate temperatures (75–100 °C). The as-produced MgH_2_–ZrTi particles were observed by SEM to have irregular shapes (Fig. 1a) [26], with about 90% of the particles distributed in the range of 100–500 nm and a very small fraction up to 1 μm (Fig. S1a). No detectable variation was observed in the morphology or size distribution after the deposition of amorphous Al_2_O_3_ (MgH_2_–ZrTi@10nmAl_2_O_3_, Fig. 1b), indicating no melting or growth of MgH_2_ particles during the ALD process. Furthermore, no difference was observed in the X-ray diffraction (XRD) patterns of MgH_2_–ZrTi before and after the coating of amorphous Al_2_O_3_ (Fig. 1c). Only diffraction peaks at 27.9°, 35.7°, 39.9°, 54.6°, and 68.8°, corresponding to the (110), (101), (200), (211), and (112) planes of MgH_2_, were detected from the XRD patterns of MgH_2_–ZrTi and MgH_2_–ZrTi@10nmAl_2_O_3_ (Fig. 1c, ♥). No dehydrogenation or oxidation product was detected from the XRD of MgH_2_–ZrTi@10nmAl_2_O_3_, further confirmed no negative reaction occurred during ALD processing for hydrogen storage materials. No diffraction peak of Al_2_O_3_ was observed from MgH_2_–ZrTi@10nmAl_2_O_3_, suggesting the amorphous features of Al_2_O_3_ from the ALD process. This situation is consistent well with reported metal oxides prepared by ALD [27, 28], where no XRD signal of deposited metal oxides was detected. Only interplanar spacings of 0.25 nm corresponding to the (101) planes of MgH_2_ (JCPDS No. 12-0697) were observed from the HRTEM images of MgH_2_–ZrTi@10nmAl_2_O_3_ (Fig. S2), consistent well with XRD results. The successful coating of Al_2_O_3_ shells was clearly observed by HAADF-STEM with corresponding elemental analyses on MgH_2_–ZrTi@10nmAl_2_O_3_ (Fig. 1d). The Al and O elements were observed to be clearly distributed as thin atomic layers on the surface of MgH_2_ particles, where Zr, Ti, and C were detected to be homogeneously distributed inside MgH_2_ particles as hydrogen channels [26].

### Shied Effect of Amorphous Al_2_O_3_ Coating on MgH_2_–ZrTi

**Fig. 2:**
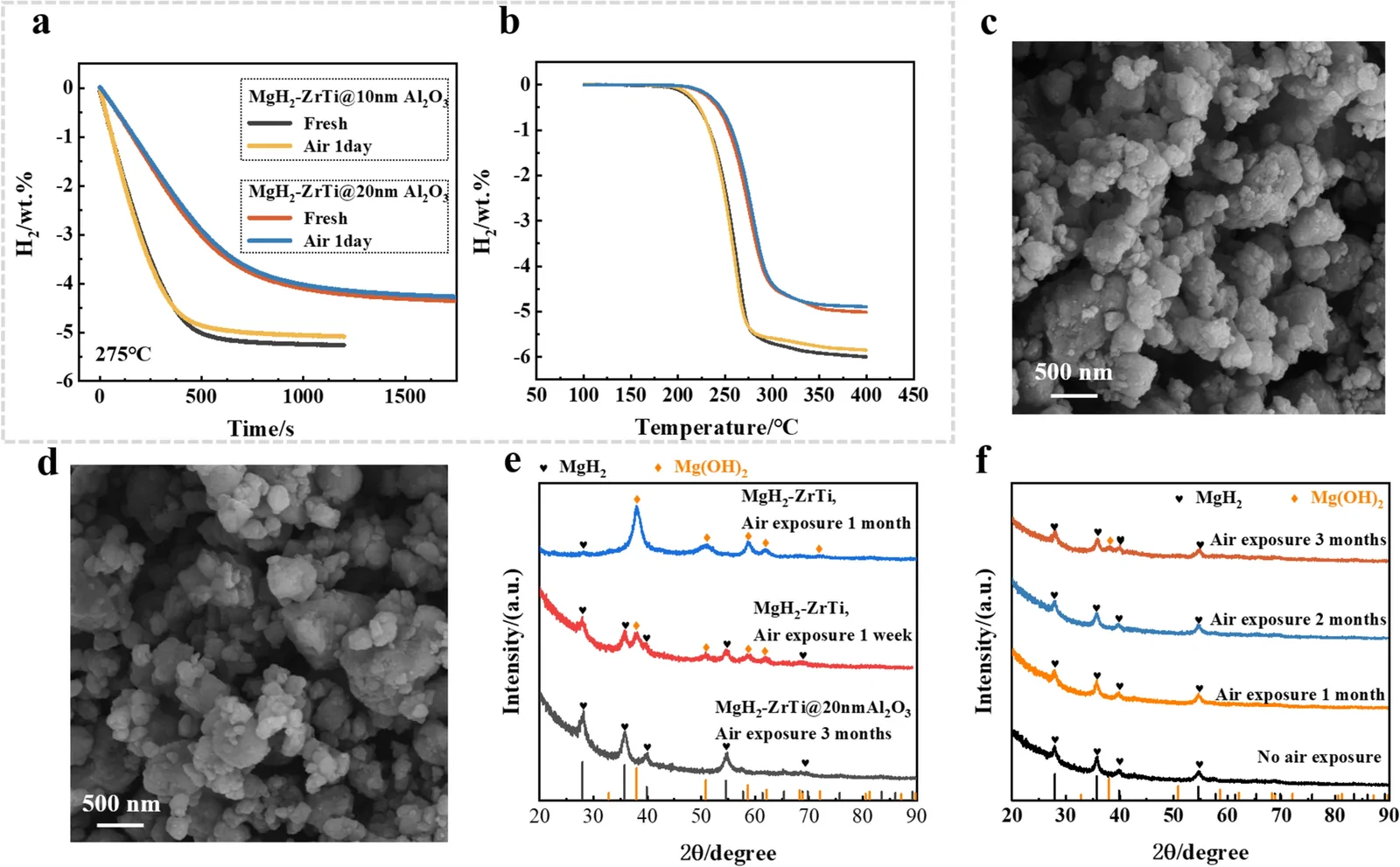
**a** Isothermal dehydrogenation (275 °C) and **b** TPD curves of MgH_2_–ZrTi@10nmAl_2_O_3_ (fresh–black, exposure to air for 1 day–yellow) and MgH_2_–ZrTi@20nmAl_2_O_3_ (fresh–red, exposure to air for 1 day–blue) before and after exposure to air for 1 day. SEM images of MgH_2_–ZrTi@10nmAl_2_O_3_ after exposure to air for **c** 1 month and **d** 2 months. XRD spectra of **e** MgH_2_–ZrTi, MgH_2_–ZrTi@20nmAl_2_O_3_, and **f** MgH_2_–ZrTi@10nmAl_2_O_3_ before and after air exposure for different times

The isothermal (Fig. 2a) and temperature programmed dehydrogenation (TPD, Fig. 2b) properties of MgH_2_–ZrTi@10nmAl_2_O_3_ and MgH_2_–ZrTi@20nmAl_2_O_3_ before and after exposure to air were measured to reveal the shielding effect of amorphous Al_2_O_3_ shells to H_2_O and O_2_. The dehydrogenation performance of MgH_2_–ZrTi was found to deteriorate significantly after exposure to air for 1 day (Fig. S3). Only 0.036 wt% of H_2_ was detected to be released from MgH_2_–ZrTi after exposure to air for 1 day at 275 °C in 20 min, while 6.20 wt% H_2_ was detected at 275 °C in 6 min from MgH_2_–ZrTi before exposure to air (Fig. S3a). The dehydrogenation temperature of MgH_2_–ZrTi was found to be significantly increased after exposure to air for 1 day, where only 1.10 wt% of H_2_ was released even up to 400 °C compared to 6.40 wt% released up to 300 °C for fresh MgH_2_–ZrTi from TPD measurements (Fig. S3b). However, no significant variation was detected for the dehydrogenation of MgH_2_–ZrTi@Al_2_O_3_ (Fig. 2a, b), where less than 0.2 wt% variation was observed for MgH_2_–ZrTi@Al_2_O_3_ after exposure to air for 1 day. The dehydrogenation curves of MgH_2_–ZrTi@Al_2_O_3_ before and after air exposure were found to overlap for the isothermal one within 0–400 s (Fig. 2a) as well as TPD one within 100–280 °C (Fig. 2b). About 4.87 wt% of H_2_ was released at 275 °C within 500 s from MgH_2_–ZrTi@10nmAl_2_O_3_ after exposure to air for 1 day (Fig. 2a). The hydrogen storage density was found to decrease with increasing Al_2_O_3_ thickness from 10 to 20 nm (Fig. 2a, b). About 4.23/4.31 wt% H_2_ was released at 275 °C within 25 min for MgH_2_–ZrTi@20nmAl_2_O_3_ before/after air exposure (Fig. 2a). The dehydrogenation temperature of MgH_2_–ZrTi@Al_2_O_3_ was also found to increase with increasing Al_2_O_3_ thickness (Fig. 2b). The ZrO_2_ and Ti_3_C_2_ components of MgH_2_–ZrTi composites were deduced from the maximum dehydrogenation capacity of fresh MgH_2_–ZrTi (6.77 wt%, TPD in Fig. S3b) to have a mass ratio of about 11 wt%.

The XRD features of Mg(OH)_2_ (JCPDS NO. 07-0239, Fig. 2e, yellow ♦) was clearly detected in addition to XRD features of MgH_2_ (27.9°(110), 35.7°(101), 39.9°(200), 54.6°(211), and 68.8°(112), Fig. 2e, black ♥) for MgH_2_–ZrTi after exposure to air, whose intensity was found to increase with increasing exposure time (Fig. 2e from red line to blue line), consistent well with the degradation of its hydrogen storage performance (Fig. S3). In order to estimate Mg(OH)_2_ impurity quantitatively, the MgH_2_–ZrTi (100 mg) was heated at 200 °C for 48 h in air after exposure to air for 1 week. The mass of the product was measured to be 181.2 mg. Both MgH_2_ and Mg(OH)_2_ (Fig. 2e, red line) was detected in MgH_2_–ZrTi before heating treatment, while only Mg(OH)_2_ (Fig. S4) was detected after heating treatment. The MgH_2_ and Mg(OH)_2_ in the MgH_2_–ZrTi after exposure to air for 1 week was estimated to be about 66 and 34 mg, respectively, based on the mass variation. The Mg(OH)_2_ impurities in MgH_2_–ZrTi after exposure to air for 1 week was deduced to be about 34%. No Mg(OH)_2_ was detected from the XRD of MgH_2_–ZrTi@5nmAl_2_O_3_ after exposure to air for 1 week (Fig. S5), demonstrating that the 5 nm amorphous Al_2_O_3_ encapsulation is also effective in protecting MgH_2_/Mg particles. However, the Mg(OH)_2_ features were detected after exposure to air for 1 month and 6 weeks, with its content increasing continuously (Fig. S5). However, only diffraction features of MgH_2_ were detected for MgH_2_–ZrTi@10nmAl_2_O_3_ and MgH_2_–ZrTi@20nmAl_2_O_3_ even after exposure to air for 2 to 3 months (Fig. 2e, f), where no MgO or Mg(OH)_2_ was detected. Only tiny diffraction peaks of Mg(OH)_2_ were detected from MgH_2_–ZrTi@10nmAl_2_O_3_ after exposure to air for 3 months (Fig. 2f, red line). No morphological changes were observed on the surface of MgH_2_–ZrTi@10nmAl_2_O_3_ particles after exposure to air for 1 month (Fig. 2c) and even 2 months (Fig. 2d). No Mg(OH)_2_ was detected from MgH_2_–ZrTi@10nmAl_2_O_3_ after exposure to air for 3 weeks when the air condition was adjusted from 15 °C to 25% RH to 30 °C and 50% RH (Fig. S6), further confirmed the air stability of the MgH_2_–ZrTi@10nmAl_2_O_3_. No diffraction peaks of MgO and Mg(OH)_2_ was detected from the Mg–ZrTi@10nmAl_2_O_3_ in non-hydrogen state after exposure to air for 3 weeks (Fig. S7) either, demonstrating an excellent protection effect of 10 nm amorphous Al_2_O_3_ shells for the hydrogen storage particles. The MgH_2_–ZrTi@5nmAl_2_O_3_, MgH_2_–ZrTi@10nmAl_2_O_3_ and MgH_2_–ZrTi@20nmAl_2_O_3_ have been demonstrated to maintain intact without obvious oxidation after exposure to air for 1 week, 2 months and 3 months, respectively. The 10 nm Al_2_O_3_ coating has been demonstrated to be effective enough to prevent the degradation of MgH_2_–ZrTi from exposure to air (H_2_O and O_2_).

### Hydrogen Selective Adsorption of MgH_2_–ZrTi@10nmAl_2_O_3_ in the Presence of CH_4_, O_2_, N_2_ and CO_2_ Impurities

**Fig. 3:**
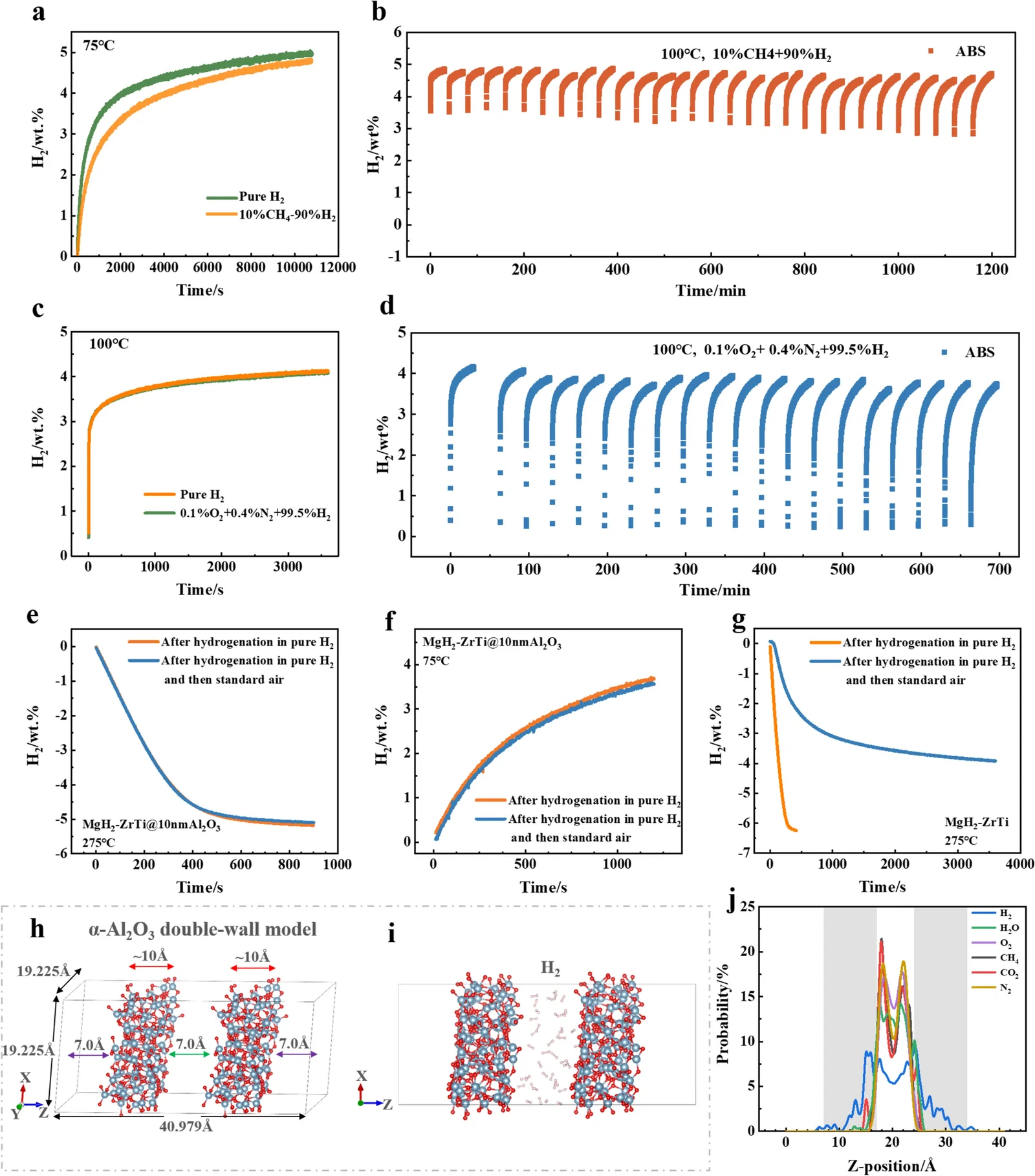
Isothermal adsorption curves of **a** MgH_2_–ZrTi@10nmAl_2_O_3_ at 75 °C in pure H_2_ and 10%CH_4_ + 90%H_2_ atmospheres (30 bar). **b** Isothermal cyclic adsorption curves of MgH_2_–ZrTi@10nmAl_2_O_3_ at 100 °C under 10%CH_4_ + 90%H_2_ atmosphere. **c** Isothermal adsorption curves of MgH_2_–ZrTi@10nmAl_2_O_3_ in pure H_2_ and 0.1%O_2_ + 0.4%N_2_ + 99.5%H_2_ atmospheres (16 bar, 100 °C). **d** Isothermal cyclic adsorption curves of MgH_2_–ZrTi@10nmAl_2_O_3_ at 100 °C under 0.1%O_2_ + 0.4%N_2_ + 99.5%H_2_ atmosphere. Isothermal **e** dehydrogenation and **f** re-hydrogenation curves of MgH_2_–ZrTi@10nmAl_2_O_3_ after hydrogenation in pure H_2_ followed 21%O_2_ + 79%N_2_ (blue line) at 75 °C for 1 h compared to that only under pure H_2_ (orange line). **g** Isothermal dehydrogenation curves (275 °C) of MgH_2_–ZrTi after hydrogenation in pure H_2_ (orange line) as well as hydrogenation in pure H_2_ followed by 21%O_2_ + 79%N_2_ (blue line) at 75 °C for 1 h. **h** Double-wall α- Al_2_O_3_ model consisting of two parallel slabs and **i** the simulation of the penetration of H_2_ molecules between the two Al_2_O_3_ slabs. **j** Proportional distribution of various gas molecules along the z-direction

The selective absorption of MgH_2_–ZrTi@10nmAl_2_O_3_ was first explored under a hydrogen atmosphere in the presence of 10% methane (10 vol%CH_4_ + 90 vol%H_2_). About 4.79 wt% H_2_ was detected to be adsorbed by MgH_2_–ZrTi@10nmAl_2_O_3_ under 10%CH_4_ + 90%H_2_ (30 bar), slightly less than that (5.00 wt%) under pure H_2_ (30 bar, 99.999%) at 75 °C in 3 h (Fig. 3a). The absorption densities under two different atmospheres were observed to converge with increasing absorption time (Fig. 3a). The slightly slower hydrogenation kinetic under an impure hydrogen atmosphere than that under a pure hydrogen atmosphere was attributed to lower real-time hydrogen pressure of the impure atmosphere than that of pure one during the hydrogenation process, which is confirmed by the overlap of the hydrogenation curve of MgH_2_–ZrTi@10nmAl_2_O_3_ under 30 bar pure hydrogen and 35 bar 10%CH_4_ + 90%H_2_ (Fig. S8). Strong MgH_2_ feature (Fig. S9, black line, black ♥) with very weak Mg feature (Fig. S9, black line, orange *) were detected from hydrogenated MgH_2_–ZrTi@10nmAl_2_O_3_ under impure hydrogen atmosphere (10%CH_4_ + 90%H_2_), consistent well with its hydrogenation results (Fig. 3a, yellow). Only a slight difference, slower hydrogenation kinetics, has been demonstrated for MgH_2_–ZrTi@10nmAl_2_O_3_ under an impure hydrogen atmosphere (10%CH_4_ + 90%H_2_) compared to that under a pure hydrogen atmosphere (99.999%). However, the MgH_2_–ZrTi without Al_2_O_3_ shells was detected to have a much lower absorption density under 10%CH_4_ + 90%H_2_ than that under a pure hydrogen atmosphere (Fig. S10). About 4.71 wt% H_2_ was adsorbed by MgH_2_–ZrTi in 1 h under pure H_2_ atmosphere (Fig. S10, green line), whereas about 3.91 wt% was absorbed under 10%CH_4_ + 90%H_2_ atmosphere (Fig. S10, blue line). The adsorption capacity of MgH_2_–ZrTi in 10%CH_4_ + 90%H_2_ atmosphere was detected to decay gradually with increasing cycles, with 3.48 wt% as well as 3.18 wt% adsorbed within 1 h for the 2nd and 3rd cycles, respectively (Fig. S10, orange and red lines). Fish scale-like structures (Fig. S11b), amorphous carbon produced from the reaction of CH_4_ with MgH_2_–ZrTi particles [25], were detected to emerge on the surface of MgH_2_–ZrTi after hydrogenation under 10%CH_4_ + 90%H_2_ atmosphere. The emergence of carbon was confirmed by HAADF-STEM and its elemental analysis of MgH_2_–ZrTi before and after hydrogenation under10%CH_4_ + 90%H_2_ atmosphere, where the carbon range was enhanced after hydrogenation (inside the yellow circle of Figs. S12 and S13). Only XRD features of MgH_2_ and Mg were detected from the hydrogenated MgH_2_–ZrTi (10%CH_4_ + 90%H_2_, Fig. S9), indicating the amorphous nature of carbon which is consistent well with reported data [25].

The isothermal adsorption performance of MgH_2_–ZrTi@10nmAl_2_O_3_ was further measured at different temperatures under 10%CH_4_ + 90%H_2_ atmosphere (Fig. S14a), where 3.86 wt% H_2_, 5.33 wt% H_2_, and 5.85 wt% H_2_ were absorbed at 75, 100, and 125 °C within 1 h. The MgH_2_–ZrTi@10nmAl_2_O_3_ was observed to reach the inflection point of the adsorption curve at around 4 min at 100 and 125 °C, with 4.33 and 4.84 wt% H_2_ absorbed, respectively. No Fish scale-like structures of amorphous carbon were observed on the surface of MgH_2_–ZrTi@10nmAl_2_O_3_ after hydrogenation at 125 °C under 10%CH_4_ + 90%H_2_ atmosphere from SEM (Fig. S15). No amorphous carbon produced was further confirmed by HAADF-STEM and corresponding elemental analyses (Fig. S16), where no extra carbon was observed on Al_2_O_3_ shells. The selective hydrogen absorption of MgH_2_–ZrTi@10nmAl_2_O_3_ was further confirmed by its cycling stability under 10%CH_4_ + 90%H_2_ atmosphere (Figs. S14b and S17). The 1st and 5th adsorption curves of MgH_2_–ZrTi@10nmAl_2_O_3_ were observed to be almost completely overlapped. About 3.98 and 3.89 wt% H_2_ was absorbed at 75 °C in 1 h for the 1st and 5th cycle, respectively (Fig. S14b). About 4.86, 4.72, and 4.71 wt% H_2_ were still absorbed by MgH_2_–ZrTi@10nmAl_2_O_3_ at 100 °C for 10th, 20th and 30th cycle, with 96.9% capacity retention at the 30th cycle (1st hydrogenation capacity was 4.86 wt%, Fig. 3b). The hydrogenation behavior that did not start from zero is attributable to the excessive rate of hydrogenation during the initial 1–3 s at 100 °C under 30 bar 10%CH_4_ + 90%H_2_ atmosphere. The MgH_2_–ZrTi@10nmAl_2_O_3_ has been demonstrated to have excellent selective hydrogenation under an impure hydrogen atmosphere of 10%CH_4_ + 90%H_2_.

The selective absorption of MgH_2_–ZrTi@10nmAl_2_O_3_ in the presence of O_2_ and N_2_ was also explored with different content. The isothermal re-hydrogenation curve of MgH_2_–ZrTi@10nmAl_2_O_3_ under 0.1%O_2_ + 0.4%N_2_ + 99.5%H_2_ (100 °C, 16 bar) was found to overlap with that under pure H_2_ (Fig. 3c), about 4.10 wt% of H_2_ was adsorbed at 100 °C within 1 h. However, the isothermal hydrogenation behavior of blank MgH_2_–ZrTi was observed to attenuate significantly under 0.1%O_2_ + 0.4%N_2_ + 99.5%H_2_, only 2.51 wt% H_2_ absorbed at 100 °C within 1 h (Fig. S18, blue line). 3.71 wt% H_2_ was absorbed by MgH_2_–ZrTi at 100 °C in 2 min under pure H_2_ at 100 °C (Fig. S18, green line). The MgH_2_–ZrTi@10nmAl_2_O_3_ was observed to have rapid hydrogenation at moderate temperatures (75–125 °C) under 0.1%O_2_ + 0.4%N_2_ + 99.5%H_2_ (Fig. S19a), where 3.15 wt% H_2_ was absorbed at 75 °C within 1 h and 3.93 wt% H_2_ was absorbed at 125 °C within 3 min. The three hydrogenation cycle curves of MgH_2_–ZrTi@10nmAl_2_O_3_ under 0.1%O_2_ + 0.4%N_2_ + 99.5%H_2_ atmosphere at 100 °C were detected to be almost overlapped (Fig. S19b). However, the cycling performance of MgH_2_–ZrTi under 0.1%O_2_ + 0.4%N_2_ + 99.5%H_2_ atmosphere at 100 °C was observed to decrease significantly (Fig. S18), with 2.52, 1.93, and 1.33 wt% of H_2_ absorbed within 1 h for the 1st, 2nd, and 3rd cycle, respectively. In addition, about 3.94 wt% as well as 3.70 wt% H_2_ were still absorbed by MgH_2_–ZrTi@10nmAl_2_O_3_ for the 10th and 20th cycle at 100 °C under 0.1%O_2_ + 0.4%N_2_ + 99.5%H_2_, with a capacity retention of 89.4% at the 20th cycle (the 1st hydrogenation capacity was 4.14 wt%, Fig. 3d). Small fluctuations in the capacity during the hydrogenation cycles (e.g., 7th and 8th hydrogenation processes, Fig. 3d) were introduced by small sample volume variation during the test [28]. No diffraction peaks of MgO (JCPDS No. 45-0946) were observed in the XRD of MgH_2_–ZrTi@10nmAl_2_O_3_ (Fig. S20, green line) after hydrogenation under 0.1%O_2_ + 0.4%N_2_ + 99.5%H_2_, where only MgH_2_ (JCPDS No. 12-0697, black ♥) and Mg (JCPDS No. 35-0821, orange *) were detected. However, MgH_2_ (JCPDS No. 12-0697, black ♥), Mg (JCPDS No. 35-0821, orange *) and MgO (JCPDS No. 45-0946, blue ∆) were observed in the XRD pattern of MgH_2_–ZrTi after hydrogenation under 0.1%O_2_ + 0.4%N_2_ + 99.5%H_2_ at 100 °C (Fig. S20, gray line). The Al_2_O_3_ shells have been demonstrated to effectively shield the MgH_2_/Mg to react with O_2_ to yield MgO, resulting in excellent selective hydrogen absorption under 0.1%O_2_ + 0.4%N_2_ + 99.5%H_2_ atmosphere. The MgH_2_–ZrTi@10nmAl_2_O_3_ has been demonstrated to have excellent hydrogen selective absorption in the presence of O_2_ and N_2_.

The selective hydrogen absorption of MgH_2_–ZrTi@10nmAl_2_O_3_ under high concentration of O_2_ and N_2_ was also performed. However, too high concentration of O_2_ with H_2_ will cause an explosion during experiments. So selective hydrogen absorption of MgH_2_–ZrTi@10nmAl_2_O_3_ with a high concentration of O_2_ was conducted by a two-step method, where the sample was first heated in pure hydrogen and then in 21%O_2_ + 79% N_2_ at 75 °C for 1 h for hydrogenation process. The isothermal re/dehydrogenation curves of MgH_2_–ZrTi@10nmAl_2_O_3_ after hydrogenation in pure H_2_ followed by 21%O_2_ + 79%N_2_ at 75 °C for 1 h were found to overlap with those after hydrogenation in pure H_2_ (Fig. 3e, f). However, the dehydrogenation curve of MgH_2_–ZrTi after being treated by pure H_2_ followed by 21%O_2_ + 79%N_2_ at 75 °C for 1 h was found to be significantly attenuated than that treated under pure hydrogen (Fig. 3g), only 2.59 wt% of H_2_ being released in 10 min at 275 °C.

Hydrogen absorption behavior in the presence of CO_2_ (0.1%CO_2_ + 0.4%N_2_ + 99.5%H_2_) was further investigated. Significant attenuation of the hydrogen absorption behavior was observed for the blank MgH_2_–ZrTi in the presence of CO_2_ (Fig. S21a). The hydrogen absorption capacity of MgH_2_–ZrTi in the H_2_ atmosphere at 100 °C was measured to be 5.25 wt% within 0.5 h (Fig. S21a, green line), while the hydrogen absorption capacities was found to decrease to 1.66 and 0.75 wt% in the first and second cycles under 0.1%CO_2_ + 0.4%N_2_ + 99.5%H_2_ atmosphere (Fig. S21a, blue and orange lines), respectively. However, the hydrogen absorption curve (Fig. S21b, blue) of MgH_2_–ZrTi@10nmAl_2_O_3_ under 0.1%CO_2_ + 0.4%N_2_ + 99.5%H_2_ atmosphere was found to overlap with that under pure H_2_ (Fig. S21b, green). The hydrogen absorption capacities of MgH_2_–ZrTi@10nmAl_2_O_3_ under 0.1%CO_2_ + 0.4%N_2_ + 99.5%H_2_ atmosphere was detected to be 4.05, 4.01, and 3.92 wt% within three cycles (Fig. S21b, blue,orange, and red lines), further confirming the effective protection of amorphous Al_2_O_3_ shells for MgH_2_/Mg from CO_2_.

To investigate the penetration behavior of various gases through amorphous Al_2_O_3_ shells, a double-wall α- Al_2_O_3_ model comprising two parallel slabs were constructed (Fig. 3h). The interlayer spacing between the slabs was set to 7 Å, and an additional 14 Å vacuum region was introduced along the z-direction to eliminate image interactions arising from periodic boundary conditions. Amorphous Al_2_O_3_ layers were generated by cleaving an α-Al_2_O_3_ (0001) slab and performing molecular dynamics (MD) simulations at 800 K using a Langevin thermostat within the NVT ensemble for 150 ps [29]. The final configuration was extracted after the system reached thermal equilibrium. Based on HRTEM observations indicating an Al-oxide layer thickness of approximately 10 nm (Fig. S2), a comparable thickness for our model was adopted. To accelerate gas penetration, 40 gas molecules were inserted between the two Al_2_O_3_ slabs for each case (Figs. 3i and S22c-h). The simulations were carried out at 800 K. Due to the elevated gas density, the interlayer spacing tends to expand under pressure. To mitigate this effect, Al and O atoms at the periodic boundaries in the xy-plane were fixed (the gray-shaded region in Fig. S22a). Subsequently, molecular dynamics (MD) simulations were performed for 250 ps using a Langevin thermostat. To monitor gas penetration, we tracked the z-position of representative atoms: H from H_2_, C from CO_2_ and CH_4_, O from H_2_O and O_2_, and N from N_2_. The penetration behavior was analyzed by plotting histograms of these atomic z-positions (Fig. 3j). The gray-shaded regions in Figs. S22a and 3j represent the spatial extent of the Al_2_O_3_ layer. It is important to note that the surface of the amorphous layer is not atomically flat. The gray region corresponded to the z-position of the outermost atoms of the amorphous Al_2_O_3_ slab. All gases except H_2_ was found to exhibit narrow probability distributions confined between the Al_2_O_3_ layers or shallowly intercalated near the surface, indicating that only H_2_ molecules are able to permeate through the amorphous Al_2_O_3_ layer, while other gas species (O_2_, H_2_O, N_2_, CH_4_, and CO_2_) are either adsorbed onto or weakly intercalated within the surface region. The MD simulations results are well consistent with reported simulation data [30]. The H_2_ permeability and selectivity are largely dependent on the size of the nanopore. The H_2_ permeability was found to increase with increasing pore size, meanwhile hampering the selectivity [30]. Based on the selective hydrogen adsorption behavior of the MgH_2_–ZrTi@10nmAl_2_O_3_ (Fig. 3a-d) and the results of MD simulations (Fig. 3j), it is inferred that the Al_2_O_3_ shells have a suitable pore size that facilitates the permeation of H_2_ as well as hinders the permeation of other gases, which in turn exhibits excellent selective adsorption properties for hydrogen.

### Hydrogen Sorption Properties of MgH_2_–ZrTi@Al_2_O_3_

**Fig. 4:**
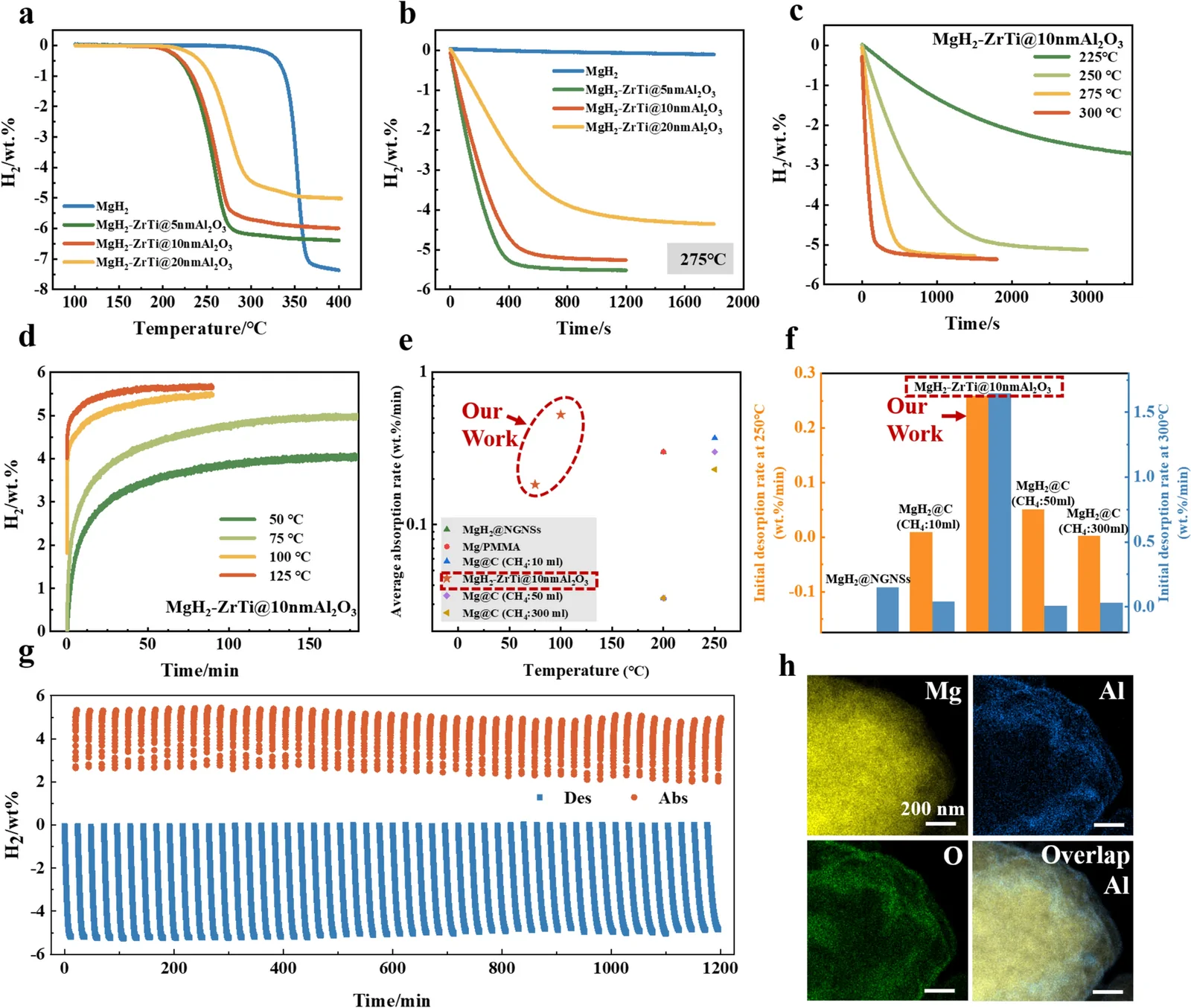
**a** TPD and **b** isothermal dehydrogenation (275 °C) of MgH_2_, MgH_2_–ZrTi@5nmAl_2_O_3_, MgH_2_–ZrTi@10nmAl_2_O_3_, and MgH_2_–ZrTi@20nmAl_2_O_3_. The isothermal **c** dehydrogenation and **d** re-hydrogenation (30 bar H_2_) of MgH_2_–ZrTi@10nmAl_2_O_3_ at different temperature. **e** Average re-hydrogenation rates and **f** Initial dehydrogenation rates (250 °C and 300 °C) of MgH_2_–ZrTi@10nmAl_2_O_3_ compared with reported excellent air-stable Mg-based composites [18–20]. **g** Isothermal dehydrogenation (275 °C/0.05 bar) and re-hydrogenation (275 °C/30 bar H_2_) cycling curves of MgH_2_–ZrTi@10nmAl_2_O_3_. **h** EDS elemental mapping analysis of MgH_2_–ZrTi@10nmAl_2_O_3_ after 50 cycles

The hydrogen sorption properties of MgH_2_ in pure H_2_ have been demonstrated to be significantly enhanced by the synergistic effect of amorphous ZrO_2_ as well as Few-layer Ti_3_C_2_ (FL-Ti_3_C_2_) in our previous studies [26]. The effects of the thickness of amorphous Al_2_O_3_ shells on the hydrogen storage performance of MgH_2_–ZrTi were also explored (Fig. 4a, b). The onset dehydrogenation temperatures of MgH_2_–ZrTi@5nmAl_2_O_3_ and MgH_2_–ZrTi@10nmAl_2_O_3_ (Fig. 4a, red and green) were found to be around 185 °C, the same as that of MgH_2_–ZrTi (Fig. S3b, orange) which is 105 °C lower than that of MgH_2_ (Fig. 4a, blue) [26]. The TPD curves of MgH_2_–ZrTi@5nmAl_2_O_3_ and MgH_2_–ZrTi@10nmAl_2_O_3_ were observed to be almost overlapped between 185 and 275 °C (Fig. 4a). However, the maximum dehydrogenation capacity of MgH_2_–ZrTi@5nmAl_2_O_3_ and MgH_2_–ZrTi@10 nm Al_2_O_3_ were observed to be 6.39 and 6.00 wt% before 400 °C (Fig. 4a), respectively, due to no hydrogen storage capacity contribution of Al_2_O_3_. The onset dehydrogenation temperature of MgH_2_–ZrTi@20nmAl_2_O_3_ was detected to increase to 205 °C (Fig. 4a, yellow). Only 4.45 wt% H_2_ was released from MgH_2_–ZrTi@20nmAl_2_O_3_ up to 300 °C, which is about 1 wt% less than that of MgH_2_–ZrTi@10nmAl_2_O_3_. The dehydrogenation kinetics of MgH_2_–ZrTi@10nmAl_2_O_3_ and MgH_2_–ZrTi@5nmAl_2_O_3_ (Fig. 4b, red and green) were found to be similar. About 5.02 wt% of H_2_ was released from MgH_2_–ZrTi@10nmAl_2_O_3_ at 275 °C within 500 s (Fig. 4b, red). The isothermal dehydrogenation rate of MgH_2_–ZrTi@20nmAl_2_O_3_ was observed to further slow down significantly (Fig. 4b, yellow), About 4.10 wt% of H_2_ was released from MgH_2_–ZrTi@20nmAl_2_O_3_ at 275 °C in 1000 s. The isothermal dehydrogenation of the hydrogen storage materials at 275 °C (Fig. 4b) were normalized based on the theoretical hydrogen storage capacity of MgH_2_ (7.6 wt%), resulting in normalized hydrogen release curves (Fig. S23). The normalized dehydrogenation curves of MgH_2_–ZrTi@5nmAl_2_O_3_ and MgH_2_–ZrTi@10nmAl_2_O_3_ within 200 s were found to almost overlap with dehydrogenation completion within 500 s, while about 1000 s was required for MgH_2_–ZrTi@20nmAl_2_O_3_ to complete the dehydrogenation process. MgH_2_–ZrTi@10nmAl_2_O_3_ has been demonstrated to be the best considering kinetics, hydrogen storage density (Fig. 4a, b), and shied effect (Figs. 2 and 3).

The dehydrogenation rates of MgH_2_–ZrTi@10nmAl_2_O_3_ were observed to be significantly accelerated with increasing temperature from 225 to 300 °C (Fig. 4c). The dehydrogenation of MgH_2_–ZrTi@10nmAl_2_O_3_ at 250 °C was found to be almost complete within 30 min, releasing about 5 wt% of H_2_ with an onset dehydrogenation rate (within the first 600 s) of 0.297 wt% min^−1^. About 5.13 wt% was released at 275 °C for 10 min with an onset dehydrogenation rate (within the first 600 s) as high as 0.683 wt% min^−1^. The dehydrogenation curve of MgH_2_–ZrTi@10nmAl_2_O_3_ was found to reach its inflection point within 3 min at a further increasing temperature to 300 °C, releasing 4.93 wt% of H_2_. Additionally, MgH_2_–ZrTi@10nmAl_2_O_3_ were hydrogenated in 30 bar 10%CH_4_ + 90%H_2_ and 16 bar 0.1%O_2_ + 0.4%N_2_ + 99.5%H_2_ for 1 h and then heated to the isothermal test temperature (250, 275, and 300 °C) under 2 bar H₂ atmosphere for isothermal dehydrogenation tests (Figs. S24a and S25a). The dehydrogenation capacity of MgH_2_–ZrTi@10nmAl_2_O_3_ after re-hydrogenation in the 10%CH_4_ + 90%H_2_ and 0.1%O_2_ + 0.4%N_2_ + 99.5%H_2_ atmospheres was slightly lower than that after re-hydrogenation in the pure H_2_ atmosphere (Fig. S26), which is attributed to the limited hydrogen absorption capacity during the hydrogen absorption process at 100 °C. An excellent hydrogenation performance was also obtained by MgH_2_–ZrTi@10nmAl_2_O_3_ (Fig. 4d). About 2.59 wt% of H_2_ was absorbed by MgH_2_–ZrTi@10nmAl_2_O_3_ at 50 °C within 15 min, extending to 3.92 wt% with hydrogenation time prolonged to 2 h. The hydrogenation capacity of MgH_2_–ZrTi@10nmAl_2_O_3_ was observed to be significantly increased at 75 °C with 3.42 wt% H_2_ absorbed within 15 min, while 4.44 wt% in 1 h and 4.97 wt% in 3 h. The surge in hydrogenation rate is attributable to the rapid enhancement of the reactivity of MgH_2_–ZrTi@10nmAl_2_O_3_ upon increasing temperature, analogous to the hydrogenation behavior of the reported blank MgH_2_–ZrTi [26]. The initial hydrogenation rate was deduced to reach 0.228 wt% min^−1^ at 75 °C based on the first 15 min since the transition point of the curve proportion limit is located at 15 min (Fig. 4d, light green). The hydrogenation curve of MgH_2_–ZrTi@10nmAl_2_O_3_ at 100 °C was found to reach its inflection point within 5 min with 4.51 wt% H_2_ absorbed, while absorbing 5.46 wt% of H_2_ within 1.5 h. The hydrogenation kinetics of MgH_2_–ZrTi@10nmAl_2_O_3_ at 125 °C (Fig. 4d, red) was found to be similar to that at 100 °C. The MgH_2_–ZrTi@10nmAl_2_O_3_ has been demonstrated to have rapid hydrogenation capabilities between 75 and 100 °C, which is able to be realized by using solar energy effectively in the future. Both the average hydrogenation rate before the transition point and initial dehydrogenation rate within the first 10 min of MgH_2_–ZrTi@10nmAl_2_O_3_ have been demonstrated to be much better than reported air-stabilized Mg-based composites under the same data-intercept conditions (Fig. 4e, f) [18–20].

The cycling performance of MgH_2_–ZrTi@10nmAl_2_O_3_ was also conducted (Fig. 4g). The dehydrogenation capacity of MgH_2_–ZrTi@10nmAl_2_O_3_ was found to gradually increase from 5.19 to 5.23 wt% during the first 10 cycles due to gradual activation of hydrogen storage material particles during the de/re-hydrogenation cycles. About 5.12 and 5.04 wt% of H_2_ were detected to release from MgH_2_–ZrTi@10nmAl_2_O_3_ even after the 20th and 30th cycles, respectively. The dehydrogenation cycling capacity of MgH_2_–ZrTi@10nmAl_2_O_3_ was found to be stable after 40 cycles (4.96 wt%), where 4.97 wt% was detected after 50 cycles. The re-hydrogenation behavior during cycling was observed to be similar to the trend of dehydrogenation one (Fig. 4g). The re-hydrogenation capacity was detected to increase from 5.30 to 5.42 wt% in the first 10 cycles. The re-hydrogenation capacity was detected to be 5.26, 4.9, 4.96, and 4.96 wt% at the 20th, 30th, 40th and 50th cycle, respectively. Amorphous Al_2_O_3_ shells were observed to be robust on the surface of MgH_2_ particles even after 50 cycles by HAADF-STEM and corresponding elemental analyses (Fig. 4h), consistent well with the high cycling performance of MgH_2_–ZrTi@10nmAl_2_O_3_. The MgH_2_–ZrTi@10nmAl_2_O_3_ has been demonstrated to have excellent cycling performance with a capacity retention of 95.0% after 50 cycles.

### Analysis of Hydrogen Sorption Processes for MgH_2_–ZrTi@10nmAl_2_O_3_

**Fig. 5:**
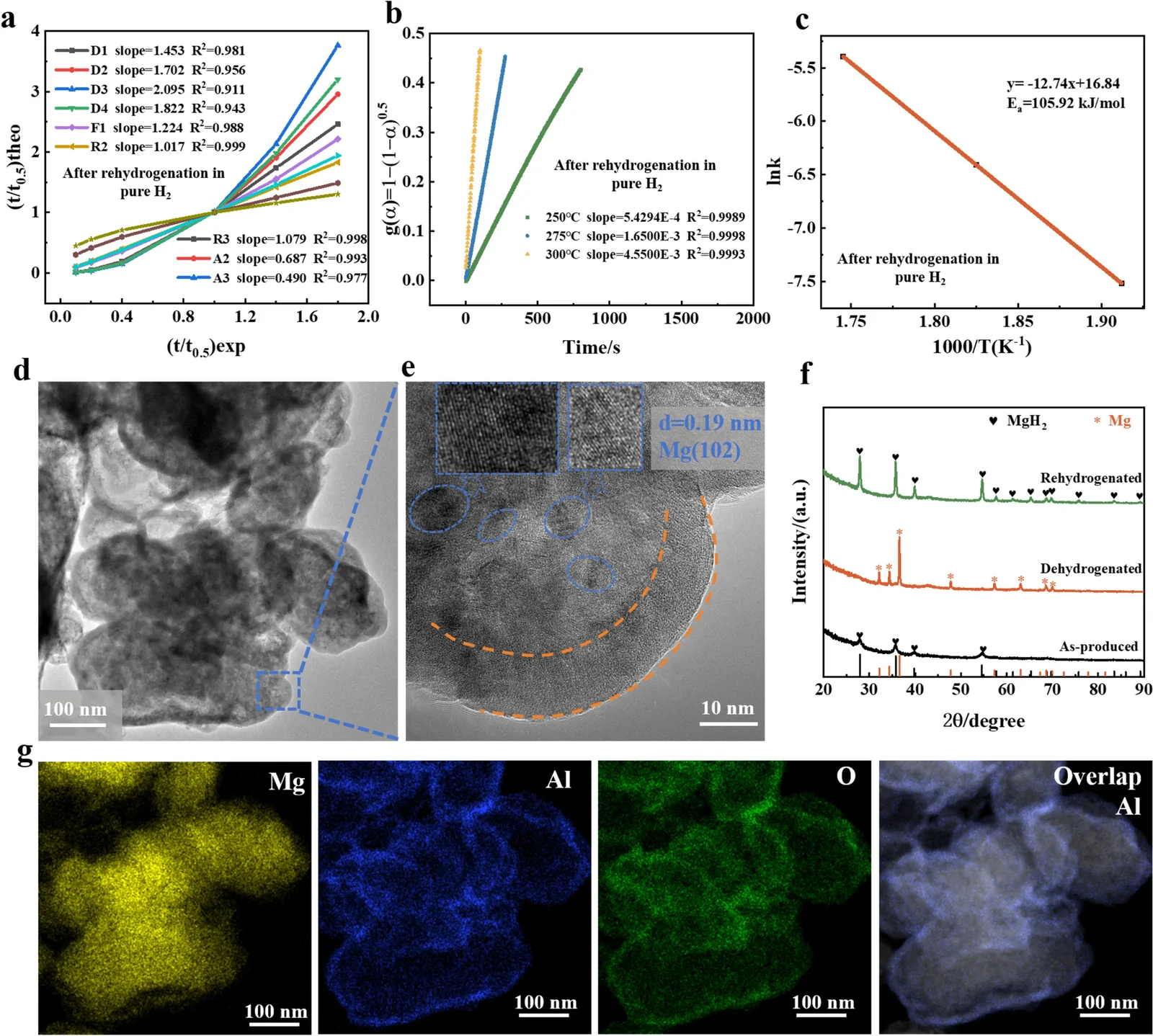
**a** Relationships of (t/t_0.5_)_theo_ vs. (t/t_0.5_)_exp_ of MgH_2_–ZrTi@10nmAl_2_O_3_ at 275 °C according to various kinetic models. **b** Time dependence of R2 modeling equation g(α) for MgH_2_–ZrTi@10nmAl_2_O_3_ at different temperatures. **c** Arrhenius plot for the dehydrogenation kinetics of MgH_2_–ZrTi@10nmAl_2_O_3_. **d** TEM, **e** HRTEM, and **g** corresponding elemental mapping analysis of MgH_2_–ZrTi@10nmAl_2_O_3_ after dehydrogenation. **f** XRD patterns of MgH_2_–ZrTi@10nmAl_2_O_3_ before and after de/re-hydrogenation

The isothermal dehydrogenation curve of MgH_2_–ZrTi@10nmAl_2_O_3_ at 275 °C (Figs. 4c, S24a, and S25a) was fitted using a rapid screening method of kinetic models [31, 32] to explore the dehydrogenation kinetic of MgH_2_–ZrTi@10nmAl_2_O_3_. The R2 (two-dimensional phase boundary) model [33] was found to suit the dehydrogenation kinetic of MgH_2_–ZrTi@10nmAl_2_O_3_ after re-hydrogenation under pure H_2_ best since its slope (1.017) is closest to 1 and intercept (− 0.0048) closest to 0 among nine kinetic models (Fig. 5a). The kinetic model of MgH_2_–ZrTi@10nmAl_2_O_3_ after re-hydrogenation in 30 bar 10%CH_4_ + 90%H_2_ or 16 bar 0.1%O_2_ + 0.4%N_2_ + 99.5%H_2_ at 100 °C for 1 h was further fitted based on the isothermal dehydrogenation data at 275 °C (Figs. S24a and S25a). The R2 model was also found to be the most suitable one for describing the dehydrogenation behavior of MgH_2_–ZrTi@10nmAl_2_O_3_ (Figs. S24b and S25b), suggesting no effect was caused by the re-hydrogenation in atmospheres containing CH_4_, O_2_, and N_2_ to the kinetics of MgH_2_–ZrTi@10nmAl_2_O_3_. Subsequently, the apparent activation energy (E_a_) for dehydrogenation was fitted based on the isothermal dehydrogenation data for MgH_2_–ZrTi@10nmAl_2_O_3_ after re-hydrogenation in 30 bar H_2_ (Fig. 4c). The isothermal dehydrogenation ratios α (at the range of 0–0.7) at different time scales under different temperatures from Fig. 4c were then substituted into the R2 model to obtain g(α)-Time curves (Fig. 5b). All three g(α)-Time curves were observed to have excellent linearity, further confirming the suitability of the R2 model for the dehydrogenation kinetic of MgH_2_–ZrTi@10nmAl_2_O_3_. The slopes of the g(α)-Time curves as well as the corresponding temperatures were substituted into the Arrhenius equation to calculate the E_a_ of the dehydrogenation of MgH_2_–ZrTi@10nmAl_2_O_3_ (Fig. 5c) [34]. The dehydrogenation activation energy E_a_ of MgH_2_–ZrTi@10nmAl_2_O_3_ was found to slightly increase to 105.92 kJ mol^−1^ from that of MgH_2_–ZrTi (E_a_ = 97.77 kJ mol^−1^) [26], consistent well with the slightly slower dehydrogenation rate of MgH_2_–ZrTi@10nmAl_2_O_3_ (Fig. 4a, b) than that of MgH_2_–ZrTi (Fig. S3) due to Al_2_O_3_ coating.

The kinetic of MgH_2_–ZrTi@10nmAl_2_O_3_ has been demonstrated to follow R2 model where re-hydrogenation under 30 bar H_2_, 30 bar 10%CH_4_ + 90%H_2_, or 16 bar 0.1%O_2_ + 0.4%N_2_ + 99.5%H_2_. The conversion reaction between MgH_2_ and Mg has been demonstrated to occur rapidly on the surface of MgH_2_ particles under the synergistic effect of amorphous ZrO_2_ and FL-Ti_3_C_2_, where effective hydrogen channels were built [26]. The H_2_ molecules were first diffused through the amorphous Al_2_O_3_ shells, with the MgH_2_/Mg interfaces formed on the surface of the hydrogen storage particles and moved to the interior of the particles through hydrogen channels at a uniform speed [34]. The movement of the MgH_2_/Mg interface was determined by the co-catalytic ability of amorphous ZrO_2_ and FL-Ti_3_C_2_ as well as the penetration rate of H_2_ molecules through amorphous Al_2_O_3_ shells, which is the key factors for the dehydrogenation kinetic of MgH_2_–ZrTi@10nmAl_2_O_3_.

The amorphous Al_2_O_3_ shells were clearly observed on the surface of MgH_2_–ZrTi@10nmAl_2_O_3_ after re/dehydrogenation by TEM as well as HRTEM images (Figs. 5d, e, and S27), consistent well with its robust features to prevent reaction of MgH_2_–ZrTi with impurities. Only the interplanar spacing of 0.19 nm corresponding to the (102) plane of Mg (JCPDS No. 35-0821) was observed from the HRTEM image of dehydrogenated MgH_2_–ZrTi@10nmAl_2_O_3_ (Fig. 5e), while only interplanar spacing of 0.25 nm corresponding to the (101) plane of MgH_2_ (JCPDS No. 12-0697) was observed from re-hydrogenated ones (Fig. S27b). The amorphous Al_2_O_3_ shells (Figs. 5e and S27) were still observed after re/dehydrogenation, further confirming their robust features during re/dehydrogenation. The amorphous feature of Al_2_O_3_ shells is well consistent with its XRD feature (Fig. 5f). The HRTEM images are consistent well with XRD spectra of MgH_2_–ZrTi@10nmAl_2_O_3_ at different stages, where only XRD pattern of Mg was observed from the dehydrogenated MgH_2_–ZrTi@10nmAl_2_O_3_ (Fig. 5f, red line) and only XRD pattern of MgH_2_ was observed from the re-hydrogenated one (Fig. 5f, green line). No XRD patterns of crystalline Al_2_O_3_ were observed from MgH_2_–ZrTi@10nmAl_2_O_3_ at any stages, further confirming its amorphous nature without any variation during re/dehydrogenation treatments. Only diffraction peaks corresponding to Mg and MgH_2_ were detected in the XRD of the de/re-hydrogenated one, indicating no detectable Mg–Al oxides were generated during heat treatments.

The chemical state variation during de/re-hydrogenation was further characterized by X-ray photoelectron spectroscopy (XPS) for MgH_2_–ZrTi@10nmAl_2_O_3_ in different states (Fig. S28). The signal at 75.9 eV corresponding to amorphous Al_2_O_3_ [35] was detected from the high-resolution Al 2*p* XPS spectrum of the as-produced MgH_2_–ZrTi@10nmAl_2_O_3_ (Fig. S28a). However, the Al 2*p* signal of MgH_2_–ZrTi@10nmAl_2_O_3_ was observed to be shifted to lower energy level by 1.23 to 74.7 eV after de/re-hydrogenation, indicating electron doping of Al after de/re-hydrogenation process. The difference in electronegativity of Al (1.61), Ti (1.54), and Zr (1.33) in MgH_2_–ZrTi@10nmAl_2_O_3_ resulted in the tendency of Al to attract electrons while Ti and Zr tended to provide electrons. Therefore, the shift in Al 2*p* spin–orbit peaks was attributed to Al-O-Ti and Al-O-Zr bonding between sublayers of Al_2_O_3_, Ti_3_C_2,_ and ZrO_2_ atoms, consistent well with reported studies [35, 36]. The high-resolution Mg 1* s* signal was only resolved into a single peak at 1303.5 eV (Fig. S28b) [37], where Mg 1* s* corresponding to Mg(OH)_2_ (1302.7 eV [38]), MgO (1303.9 eV [39]), and MgAl_2_O_4_ (1304 eV [40]) were found to be negligible. The generation of hydrolysis, oxidation, or Mg–Al-oxides phases has been demonstrated to be negligible during the de/re-hydrogenation process.

The structural stability of MgH_2_–ZrTi@10nmAl_2_O_3_ during the de/re-hydrogenation was further verified by HAADF-STEM and corresponding elemental analyses (Figs. 5g, S29, and S30). The shell-like structure composed of Al and O elements was still clearly observed on the surface of MgH_2_–ZrTi@10nmAl_2_O_3_ particles after both dehydrogenation (Fig. 5g) and re-hydrogenation (Fig. S30), further confirming the robustness of Al_2_O_3_ shells. No size variation was observed for MgH_2_–ZrTi@10nmAl_2_O_3_ particles, indicating no particle melting or growth occurred from dehydrogenation (Fig. S31) or re-hydrogenation (Fig. S32) treatments. The stable structure is particularly conducive to the stable kinetic of MgH_2_–ZrTi@10nmAl_2_O_3_, consistent well with the excellent cycling performance of MgH_2_–ZrTi@10nmAl_2_O_3_ (Fig. 4g).

## Conclusion

The atomic layer amorphous Al_2_O_3_ shells have been successfully deposited on the surface of highly active MgH_2_–ZrTi hydrogen storage material particles to obtain MgH_2_–ZrTi@Al_2_O_3_ by ALD. The Al_2_O_3_ shells have been demonstrated to be inert and effectively shielding against H_2_O, CH_4_, O_2_, N_2_, and CO_2_ while allowing H_2_ to penetrate easily, thereby achieving excellent air stability and selective hydrogen absorption performance. The dehydrogenation curves of MgH_2_–ZrTi@10nmAl_2_O_3_ were found to almost overlap before and after air exposure for 1 day, whereas almost no H_2_ was released from MgH_2_–ZrTi after exposure to air even at 275 °C. The MgH_2_–ZrTi@10nmAl_2_O_3_ was found to have excellent de/re-hydrogenation performance, with an onset dehydrogenation temperature as low as 185 °C and an absorption of 5.00 wt% of H_2_ at 75 °C within 3 h. About 5.00 wt% of H_2_ was still released from MgH_2_–ZrTi@10nmAl_2_O_3_ even after 50 cycles, with a capacity retention of up to 95%. The amorphous Al_2_O_3_ was demonstrated to be robust and maintain its shell-like structure after the de/re-hydrogenation, consistent with its high cycling stability. The MgH_2_–ZrTi@10nmAl_2_O_3_ has also been demonstrated to have high selective hydrogen absorption performance under impure hydrogen. The MgH_2_–ZrTi@10nmAl_2_O_3_ was demonstrated to have selective hydrogen adsorption in 10%CH_4_ + 90%H_2_ atmosphere (absorb 4.79 wt% H_2_ at 75 °C for 3 h), where no kinetic or density decay was observed after 30 cycles at 100 °C (96.9% capacity retention). However, the CH_4_ was detected to react with MgH_2_–ZrTi without Al_2_O_3_ shells to produce amorphous C deposition. MgH_2_–ZrTi@10nmAl_2_O_3_ has also been demonstrated to have selective hydrogen adsorption in 0.1%O_2_ + 0.4%N_2_ + 99.5%H_2_ atmosphere. About 4.1 wt% of H_2_ was absorbed by MgH_2_–ZrTi@10nmAl_2_O_3_ within 1 h at 100 °C, with a capacity retention of up to 89.4% after 20 hydrogen absorption cycles. In contrast, the hydrogen adsorption behavior of MgH_2_–ZrTi under 0.1%O_2_ + 0.4%N_2_ + 99.5%H_2_ atmosphere decayed rapidly. The MgH_2_–ZrTi@10nmAl_2_O_3_ have also been demonstrated to have excellent selective hydrogen absorption performance even with extremely high O_2_ atmosphere by two-step absorption method. The isothermal re/dehydrogenation curves of MgH_2_–ZrTi@10nmAl_2_O_3_ after hydrogenation in pure H_2_ followed by 21%O_2_ + 79%N_2_ at 75 °C for 1 h were found to overlap with those after hydrogenation in pure H_2_. In addition, about 4.0 wt% of H_2_ was absorbed by the MgH_2_–ZrTi@10nmAl_2_O_3_ at 100 °C within 0.5 h under 0.1%CO_2_ + 0.4%N_2_ + 99.5%H_2_ atmosphere, while the hydrogen absorption behavior of the MgH_2_–ZrTi was significantly attenuated under the same conditions. The MgH_2_–ZrTi@10nmAl_2_O_3_ have been demonstrated to have excellent selective hydrogen absorption performance in the presence of different amounts of CH_4_, O_2,_ N_2,_ and CO_2_.

## Supplementary Information

Below is the link to the electronic supplementary material.

Supplementary file1 (DOCX 10995 KB)
