# Supplementary material for: Solid–State Hydrogen Storage Materials with Excellent Selective Hydrogen Adsorption in the Presence of Alkanes, Oxygen, and Carbon Dioxide by Atomic Layer Amorphous Al2O3 Encapsulation
Source: Nanomicro Lett. 2025 Oct 24;18:78. doi: 10.1007/s40820-025-01934-7 (PMC12549460; doi:10.1007/s40820-025-01934-7)
Supplement: Supplementary file 1 — Supplementary file1 (DOCX 10995 KB) [file 40820_2025_1934_MOESM1_ESM.docx]

Supporting Information for

**Solid-State Hydrogen Storage Materials with Excellent Selective Hydrogen Adsorption in the Presence of Alkanes, Oxygen, and Carbon Dioxide by Atomic Layer Amorphous Al_2_O_3_ Encapsulation**

Fanqi Bu ^1^, Zhenyu Wang ^1 *^, Ali Wajid ^2^, Rui Zhai ^1^, Ting Liu ^3^, Yaohua Li ^1^, Xin Ji ^1^, Xin Liu ^1^, Shujiang Ding ^2^, Yonghong Cheng ^1^, Jinying Zhang ^1 *^

^1^ State Key Laboratory of Electrical Insulation and Power Equipment, Center of Nanomaterials for Renewable Energy (CNRE), School of Electrical Engineering, Xi'an Jiaotong University, Xi'an, Shaanxi 710049, P. R. China

^2^ School of Chemistry, Xi’an Key Laboratory of Sustainable Energy Materials Chemistry, State Key Laboratory for Mechanical Behavior of Materials, Xi’an Jiaotong University, Xi’an 710049, P. R. China

^3^ Aviation Engineering school, Air Force Engineering University, Xi’an 710038, P. R. China

*Corresponding authors. E-mail: [z.wang@mpi-susmat.de](mailto:z.wang@mpi-susmat.de) (Zhenyu Wang); [jinying.zhang@mail.xjtu.edu.cn](mailto:jinying.zhang@mail.xjtu.edu.cn) (Jinying Zhang)

**S1 Material Characterizations**

Transmission electron microscopy (TEM) images, high-resolution TEM (HRTEM) images, high-angle annular dark-field scanning transmission electron microscopy (HAADF-STEM), and elemental mapping were acquired by Lorenz Transmission Electron Microscope (Talos F200X). Scanning electron microscopy (SEM) images were recorded by Quanta 250FEG equipment. X-ray diffraction (XRD) spectra were obtained from a Bruker D2 PHASER using Cu/Kα radiation (λ =1.5418Å) at 40 kV and 30 mA. The deposited thickness of amorphous ZrO_2_/Al_2_O_3_ from ALD was measured by ellipsometry (SE401adv-C, SENTECH, Germany). The hydrogen storage properties of MgH_2_-based materials were tested using a homemade HPSA-auto apparatus [S1]. The blank MgH_2_-ZrTi used for comparison was subjected to all the sample preparation processes of MgH_2_-ZrTi@10nmAl_2_O_3_, only the ALD process without TMA pulses.

**S2 Hydrogen Sorption Measurements**

The isothermal cyclic adsorption was repeatedly performed at target temperature (75℃ or 100℃) in different atmospheres (30 bar 10%CH_4_+90%H_2_, 16 bar 0.1%O_2_+0.4%N_2_+99.5%H_2_, or 16 bar 0.1%CO_2_+0.4%N_2_+99.5%H_2_) for 0.5 h, followed by dehydrogenation treatment at 275 ℃ for 15min. The isothermal dehydrogenation of MgH_2_-ZrTi@10nmAl_2_O_3_ at different temperature (250, 275, or 300 ℃) was conducted after re-hydrogenation at 100 ℃ for 1 h under impure hydrogen atmosphere (30 bar 10%CH_4_+90%H_2_ or 16 bar 0.1%O_2_+0.4%N_2_+99.5%H_2_). Temperature programmed desorption (TPD) was tested from 100 to 400 ℃ at a heating rate of 3 ℃/min. The isothermal desorption kinetic of hydrogen storage materials was measured at different temperatures (250, 275, or 300 ℃) after re-hydrogenation under 30 bar H_2_ at different temperatures (250, 275, or 300 ℃), where the starting hydrogen pressures was set to be below 0.05 bar. The isothermal hydrogen absorption kinetic of hydrogen storage materials was tested at different temperatures (75, 100, or 125 ℃) under different atmospheres (30 bar H_2_, 30 bar 10%CH_4_+90%H_2_, 16 bar 0.1%O_2_+0.4%N_2_+99.5%H_2_, or 16 bar 0.1%CO_2_+99.9%H_2_) for 0.5h. The isothermal dehydrogenation (0.05 bar H_2_) and re-hydrogenation (30 bar H_2_) tests of the samples under pure H_2_ were performed repeatedly at 275 ℃ for the cycling measurements.

**S3 Machine Learning Molecular Dynamics**

All simulations were carried out using the LAMMPS package [S2]. Interatomic interactions were described using the Graph Atomic Cluster Expansion (GRACE) framework, specifically the GRACE-2L-OMAT model. This semi-local, two-layer machine learning potential was trained on the OMat24 dataset, which comprises over 101 million structures spanning 3.23 million materials. The dataset ensures comprehensive coverage of elemental combinations and bonding environments, with Al_2_O_3_ well-represented in both bulk and surface forms. Gas molecules such as H_2_, CH_4_, O_2_, N_2_, and CO_2_ consist of light elements (H, C, N, O) that are explicitly included in the training set and validated through Matbench Discovery benchmarks. The GRACE-2L-OMAT model has demonstrated high accuracy in predicting thermodynamic and transport properties, including thermal conductivity and diffusion behaviour (key factors in gas permeation studies). To assess the reliability of the GRACE-2L-OMAT potential for our system, we performed geometry optimizations on key molecular and solid-state structures. The results (Table S1) were consistent with density functional theory (DFT) calculations using the GGA-PBE functional, a widely accepted benchmark for structural accuracy.

**Supplementary Table and Figures**

**Table S1** Validation of GRACE-2L-OMAT via Geometry Optimization

| **System** | **Property** | **GRACE-2L-OMAT Result** | **DFT (GGA-PBE) Reference Range** |
| --- | --- | --- | --- |
| α-Al_2_O_3_ | a = b | 4.806 Å | ~4.76–4.81 Å |
|  | c | 13.134 Å | ~13.0–13.2 Å |
|  | α, β, γ | 90°, 90°, 120° | 90°, 90°, 120° |
| O_2_ molecule | O–O bond length | 1.256 Å | ~1.21–1.26 Å |
| CH_4_ molecule | C–H bond length | 1.09 Å | ~1.09 Å |
|  | H–C–H angle | 109.47° | ~109.5° |
| H_2_ molecule | H–H bond length | 0.732 Å | ~0.74 Å |
| H_2_O molecule | O–H bond length | 0.973 Å | ~0.96–0.98 Å |
|  | H–O–H angle | 105.36° | ~104.5–106° |
| CO_2_ molecule | C–O bond length | 1.178 Å | ~1.16–1.18 Å |
| **N_2_** | N–N | 1.112 Å | ~1.10–1.12 |


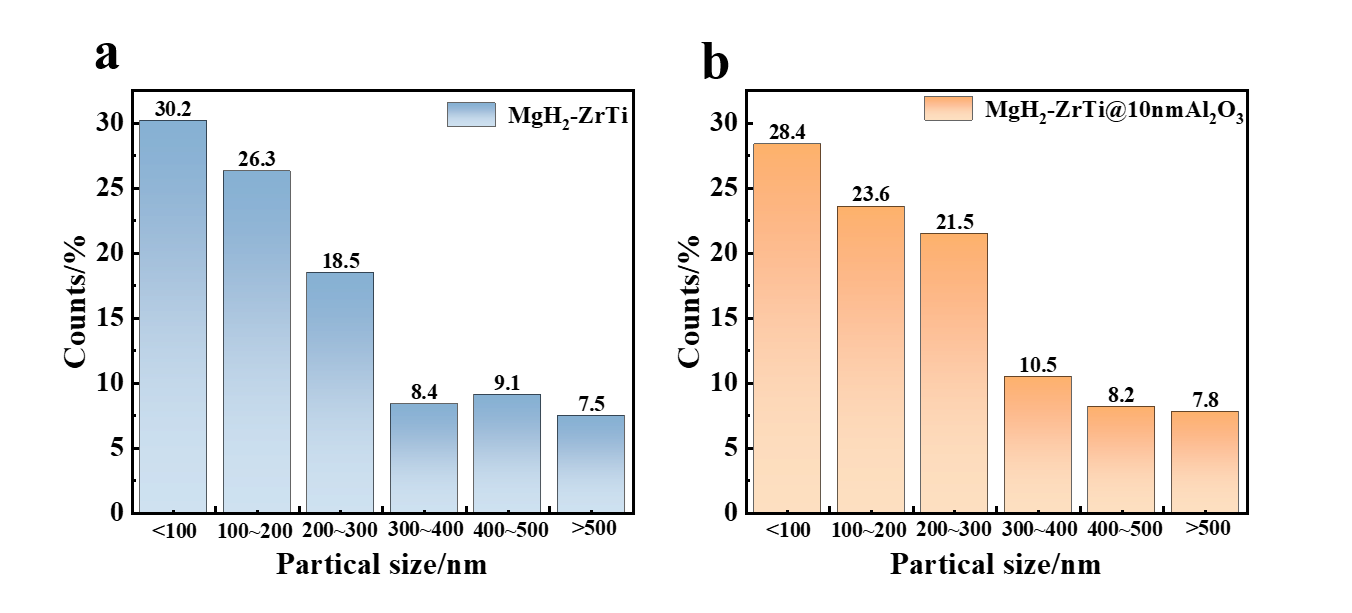


**Fig. S1** Particle size distribution of (**a**) MgH_2_-ZrTi and (**b**) MgH_2_-ZrTi@10nmAl_2_O_3_


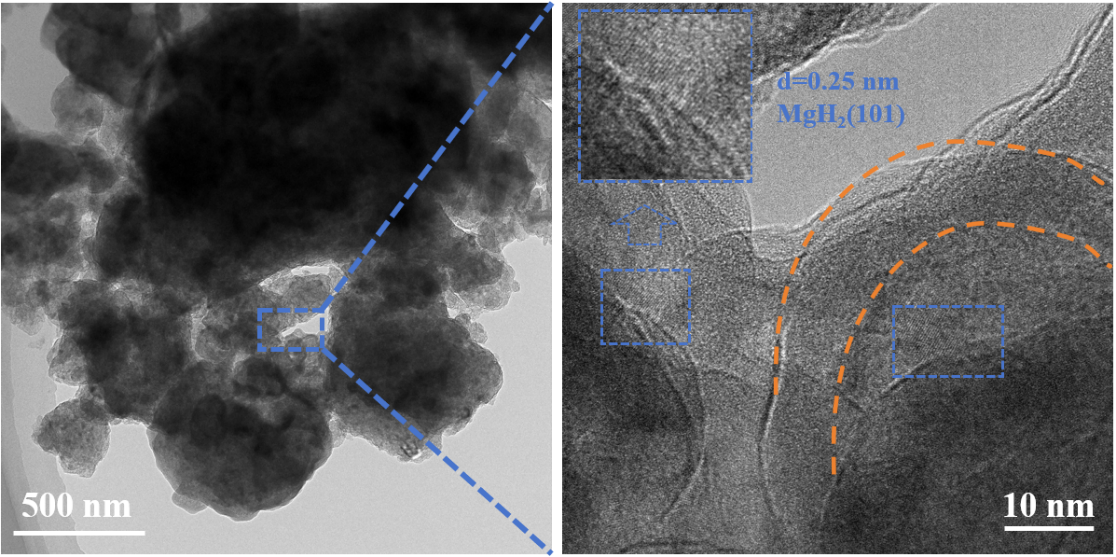


**Fig. S2** (**a**) TEM and (**b**) HRTEM of MgH_2_-ZrTi@10nmAl_2_O_3_


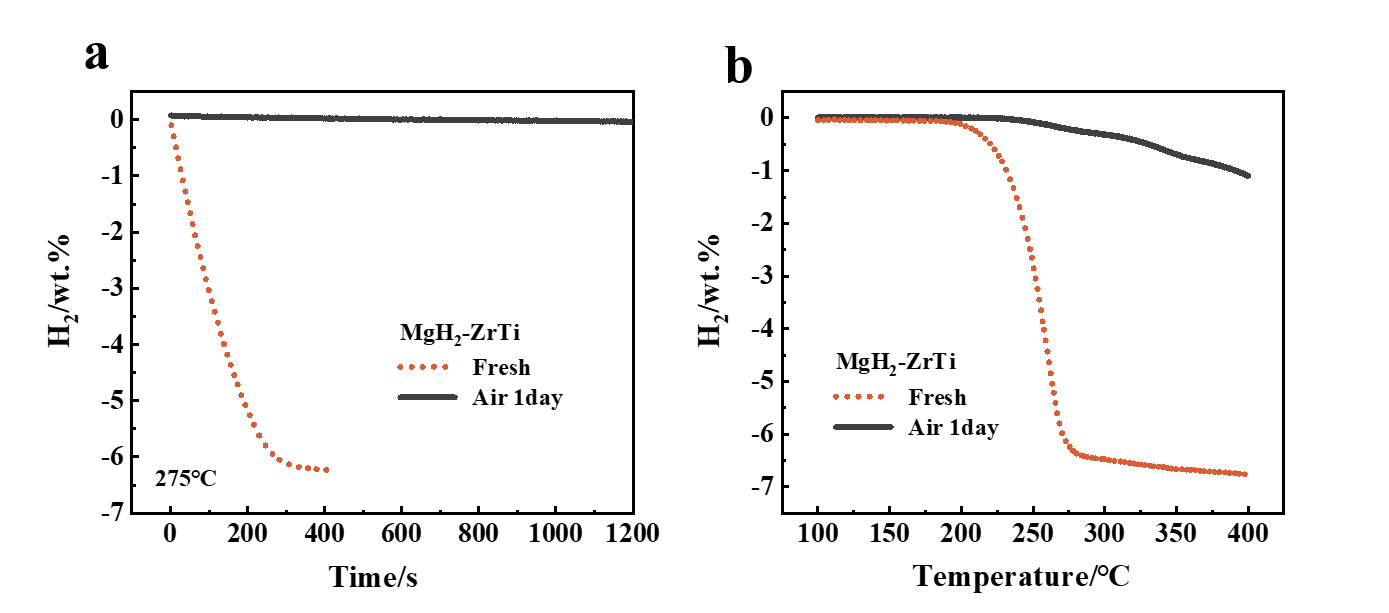


**Fig. S3** (**a**) Isothermal dehydrogenation (275 ℃) and (**b**) TPD curves of MgH_2_-ZrTi before and after exposure to air for 1 day [S3]


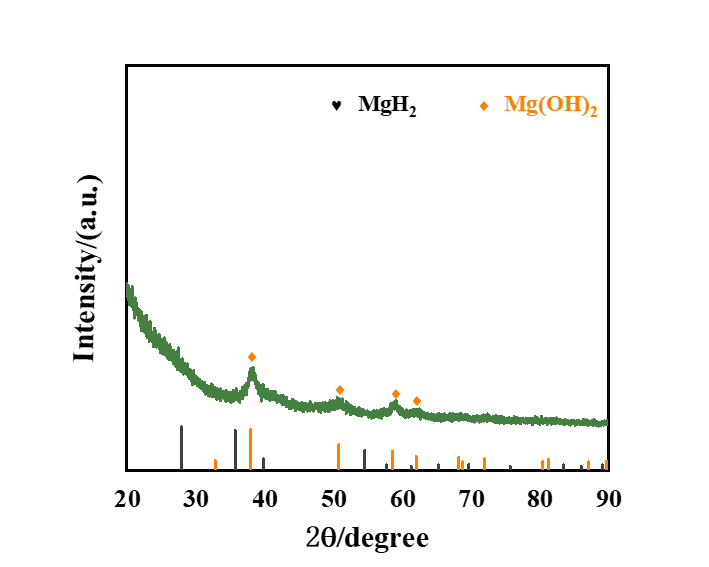


**Fig. S4** XRD patterns of MgH_2_-ZrTi after exposure to air (15 ℃ and 25% RH) for 1 week and then heated at 200℃ for 48 h in an air atmosphere


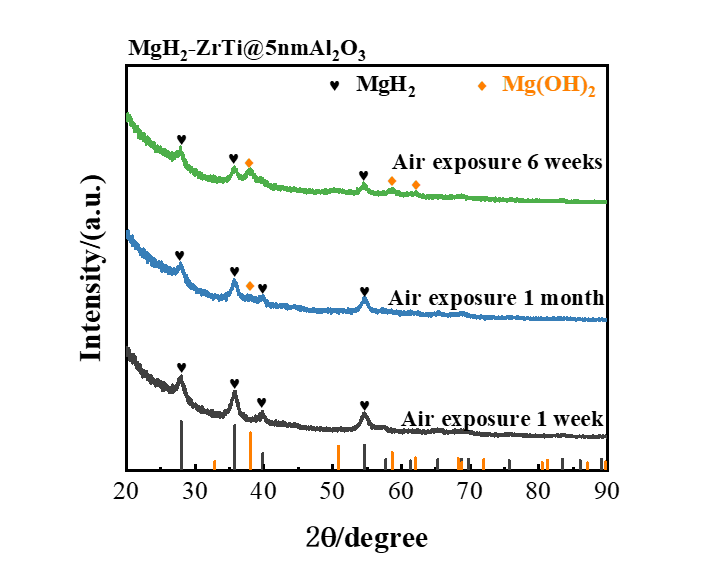


**Fig. S5** XRD patterns of MgH_2_-ZrTi@5nmAl_2_O_3_ after air exposure for different times


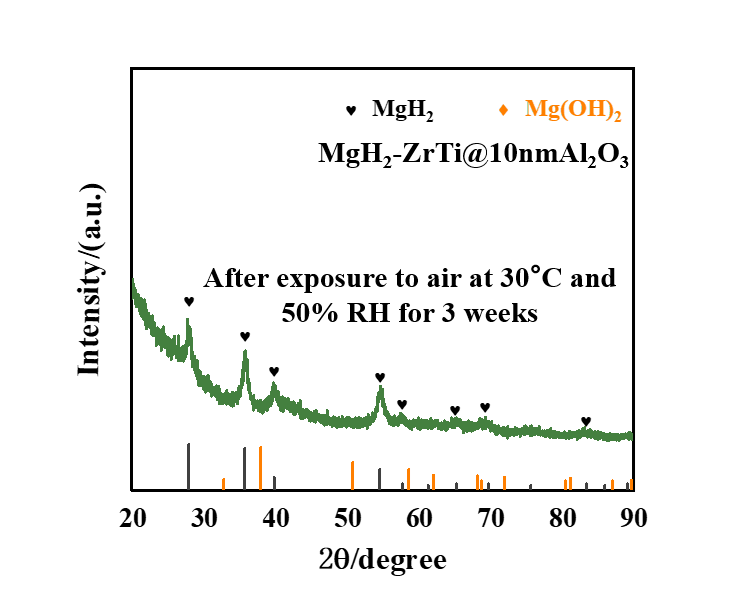


**Fig. S6** XRD pattern of MgH_2_-ZrTi@10nmAl_2_O_3_ after exposure to air at 30 ℃ and 50% RH for 3 weeks


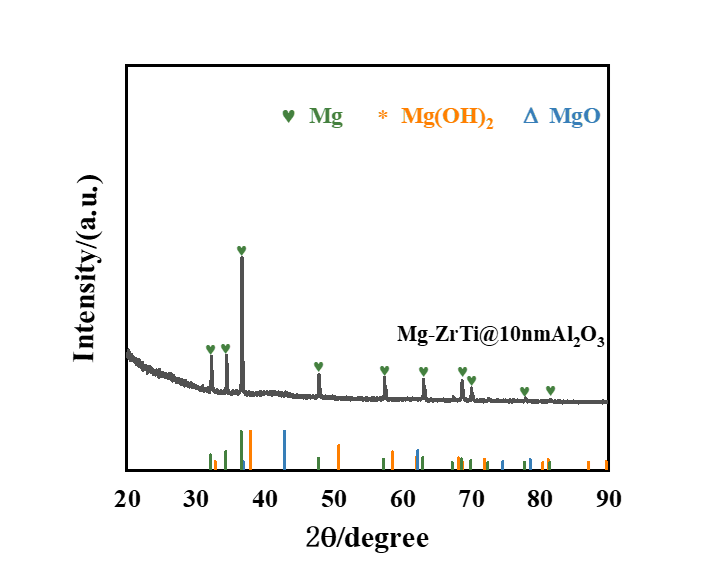


**Fig. S7** XRD patterns of Mg-ZrTi@10nmAl_2_O_3_ in non-hydrogen state after exposure to air at 30 ℃ and 50% RH for 3 weeks


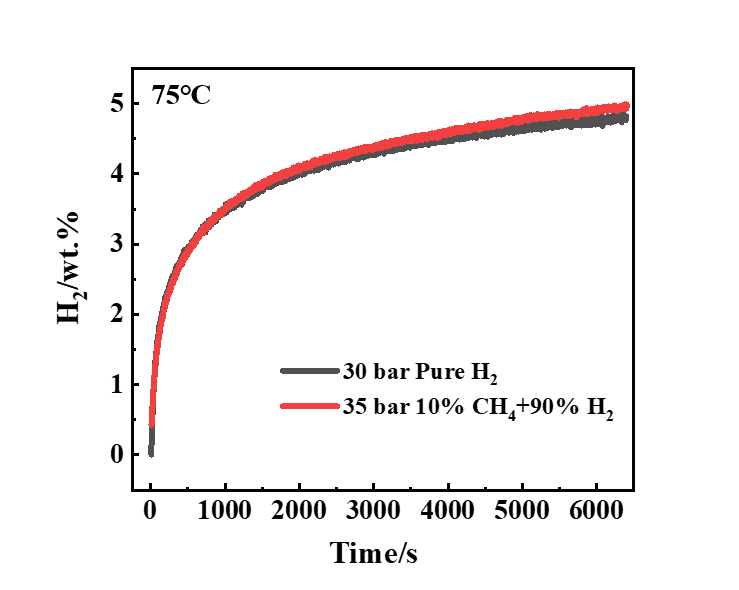


**Fig. S8** Isothermal hydrogenation curves of MgH_2_-ZrTi@10nmAl_2_O_3_ at 30 bar pure H_2_ as well as at 35 bar 10%CH_4_+90%H_2_ atmosphere


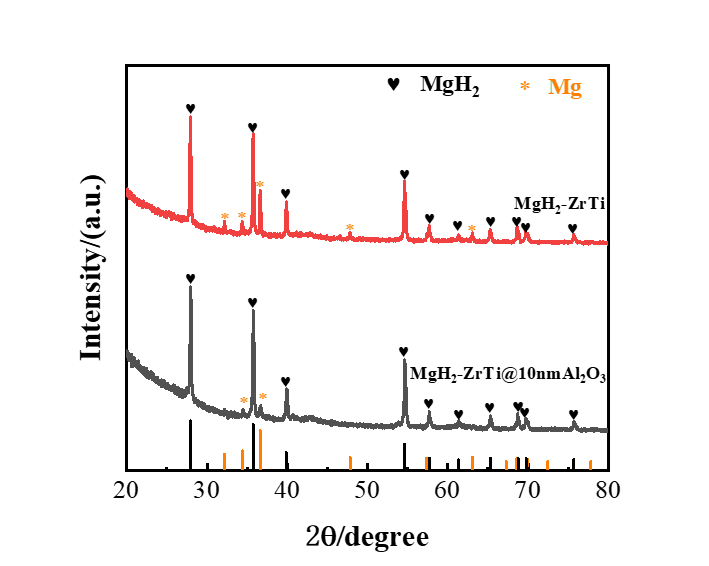


**Fig. S9** XRD patterns of MgH_2_-ZrTi (red) and MgH_2_-ZrTi@10nmAl_2_O_3_ (black) after hydrogenation in 10%CH_4_+90%H_2_ atmosphere


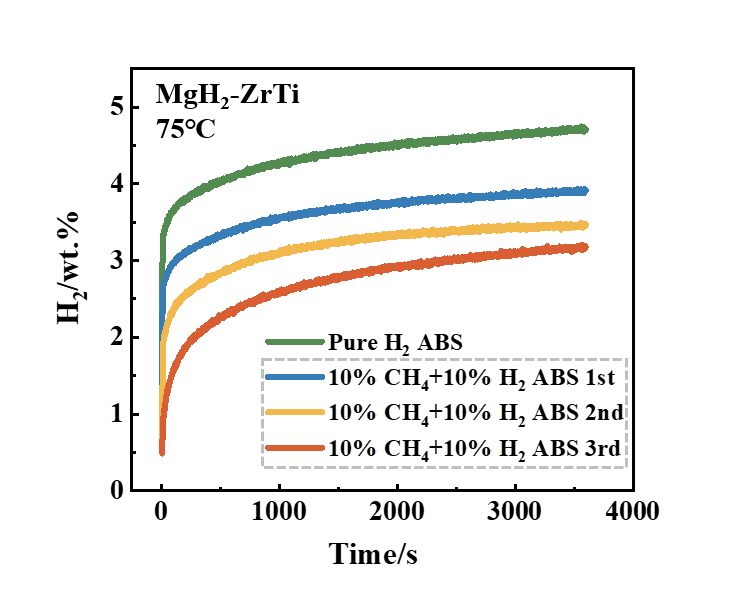


**Fig. S10** The isothermal hydrogenation curves in pure H_2_ and isothermal cyclic hydrogenation curves in 10%CH_4_+90%H_2_ atmosphere for MgH_2_-ZrTi at 75 ℃


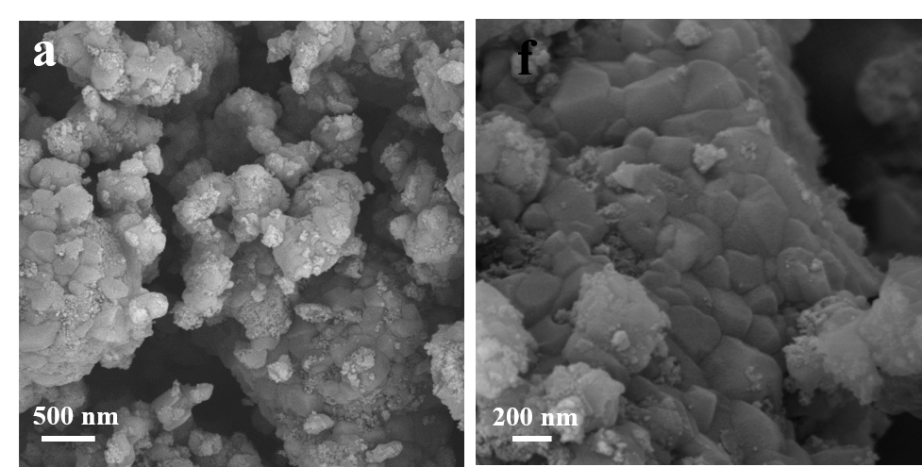


**Fig. S11** SEM image of MgH_2_-ZrTi after hydrogenation in 10%CH_4_+90%H_2_ atmosphere


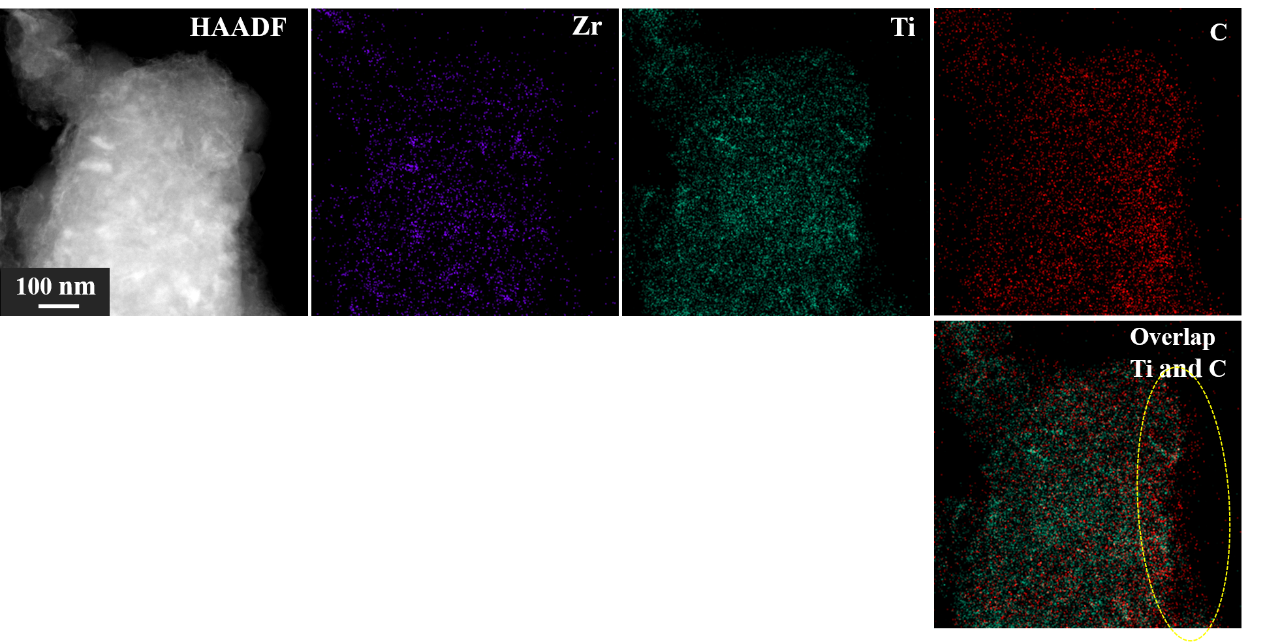


**Fig. S12** HAADF-STEM and elemental mapping analysis of blank MgH_2_-ZrTi after hydrogenation in 10%CH_4_+90%H_2_ atmosphere


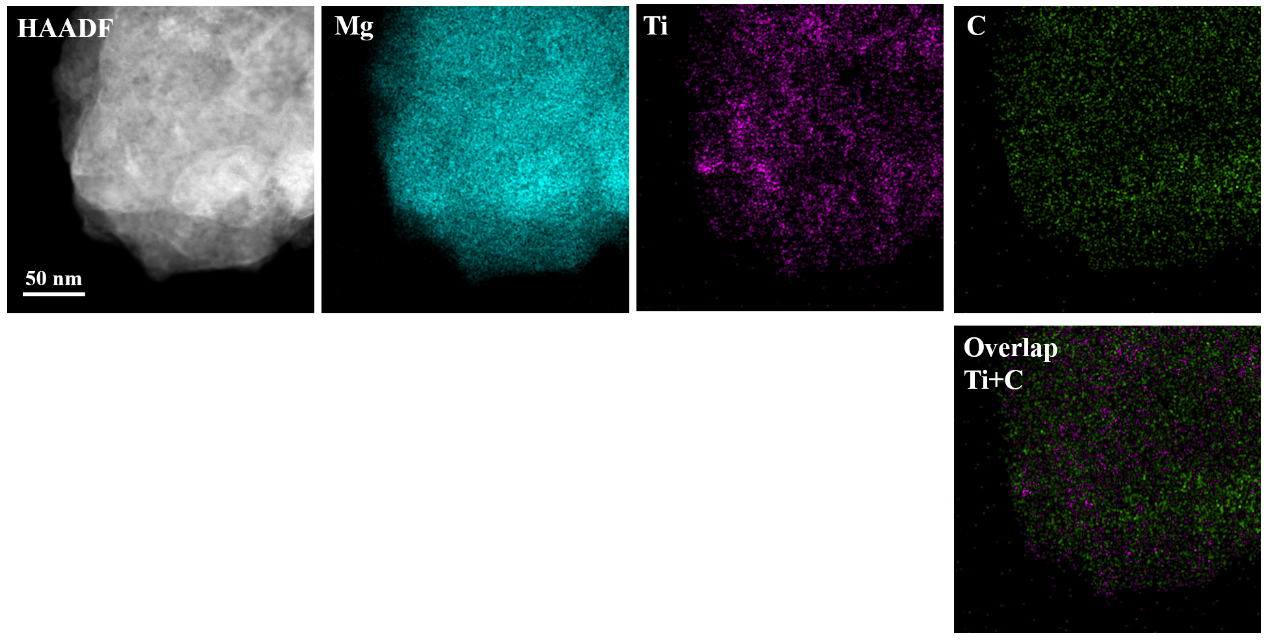


**Fig. S13** HAADF-STEM and elemental mapping analysis of MgH_2_-ZrTi before hydrogenation in 10%CH_4_+90%H_2_ atmosphere


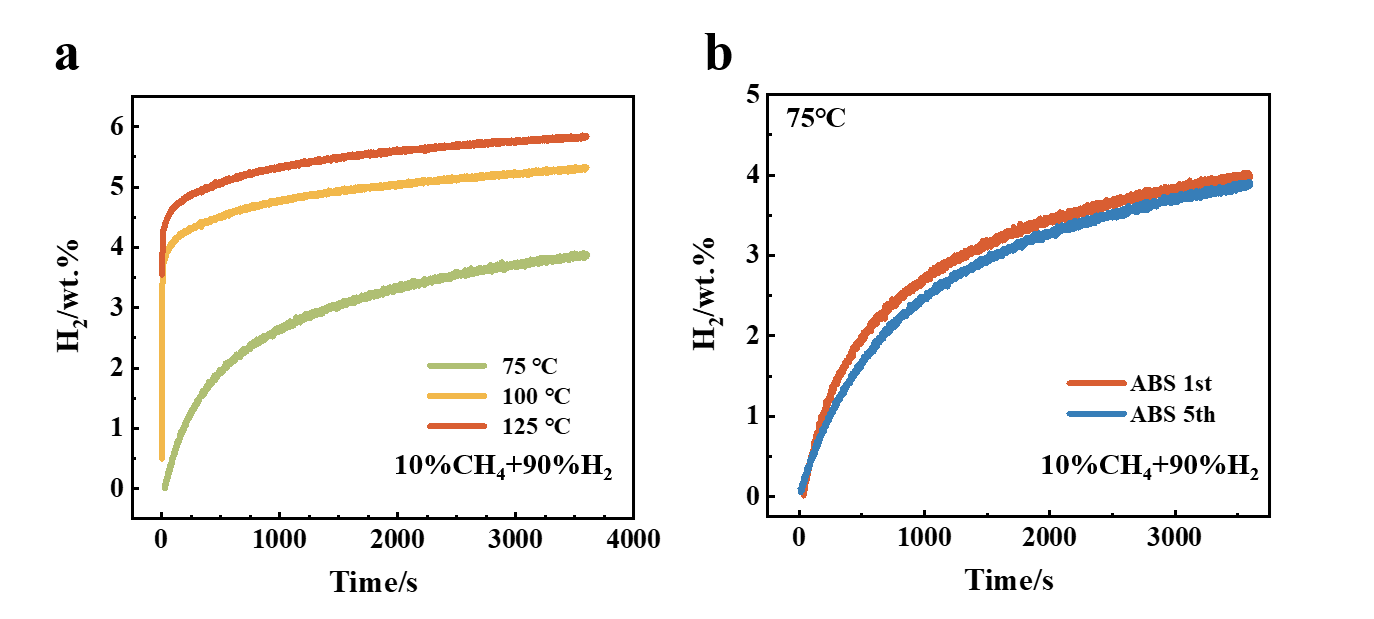


**Fig. S14** (**a**) Isothermal adsorption curves and (**b**) isothermal cyclic adsorption curves of MgH_2_-ZrTi@10nmAl_2_O_3_ under 10%CH_4_+90%H_2_ atmosphere (75 ℃)


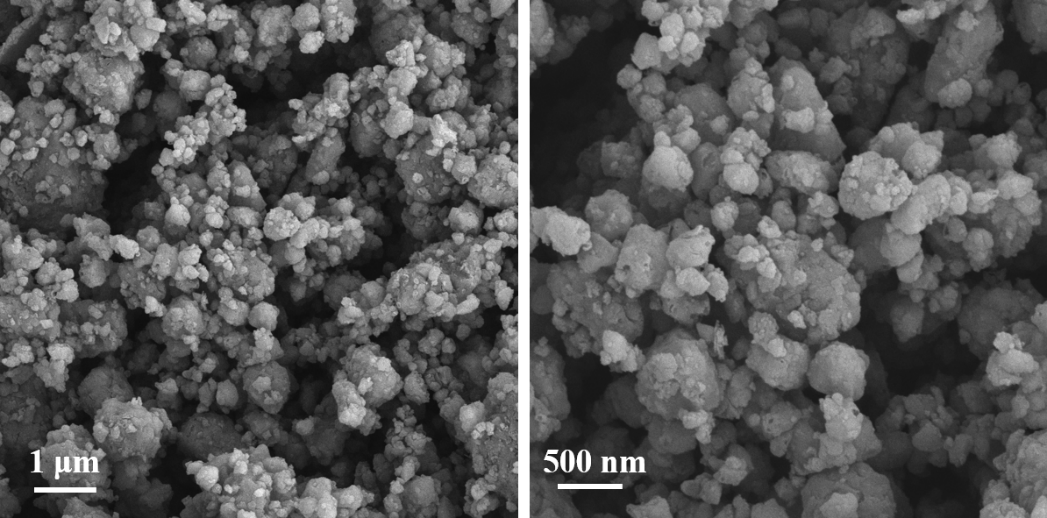


**Fig. S15** SEM images of MgH_2_-ZrTi@10nmAl_2_O_3_ after hydrogenation at 125 ℃ under 10%CH_4_+90%H_2_ atmosphere


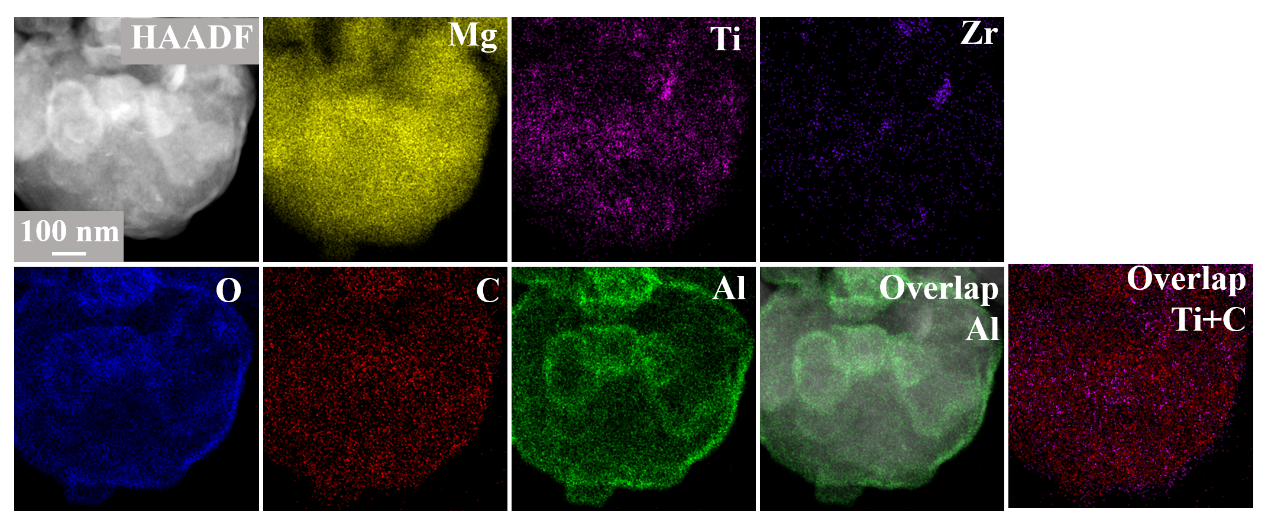


**Fig. S16** HAADF-STEM and the elemental mapping analysis of MgH_2_-ZrTi@10nmAl_2_O_3_ after hydrogenation at 125 ℃ under 10%CH_4_+90%H_2_ atmosphere


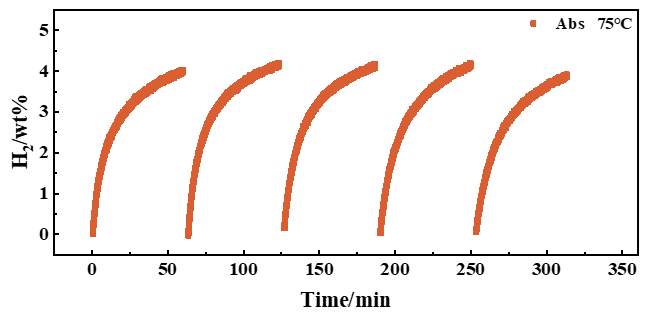


**Fig. S17** Detailed isothermal cyclic adsorption curve of MgH_2_-ZrTi@10nmAl_2_O_3_ under 10%CH_4_+90%H_2_ atmosphere


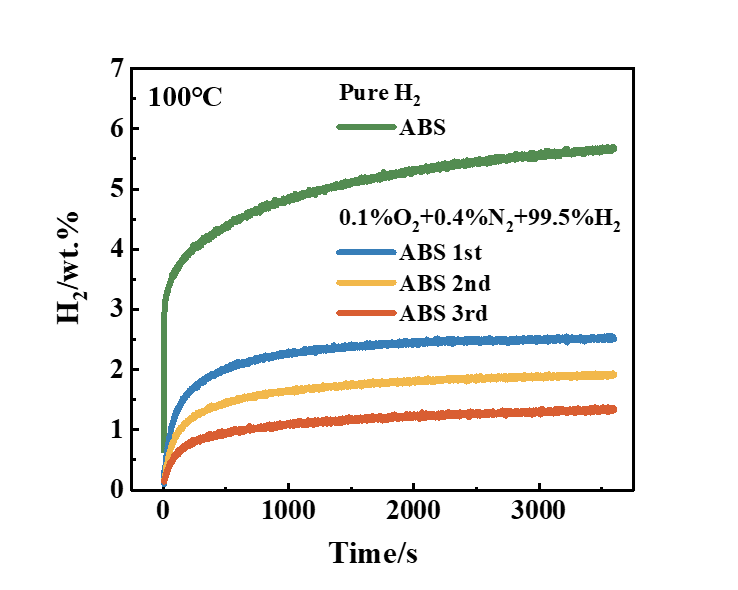


**Fig. S18** The isothermal hydrogenation curves in pure H_2_ and isothermal cyclic hydrogenation curves in 0.1%O_2_+0.4%N_2_+99.5%H_2_ atmosphere for blank MgH_2_-ZrTi at 100℃


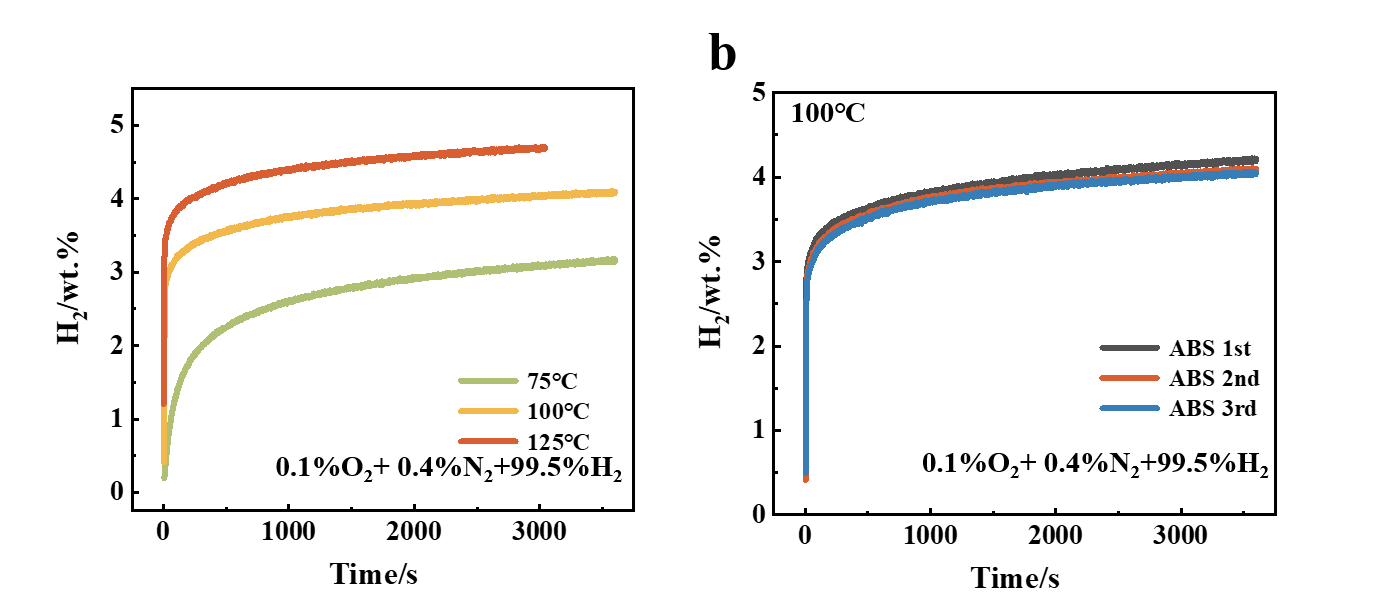


**Fig. S19** (**a**) Isothermal adsorption curves and (**b**) isothermal cyclic adsorption curve of MgH_2_-ZrTi@10nmAl_2_O_3_ under 0.1%O_2_+0.4%N_2_+99.5%H_2_ atmosphere (100 ℃)


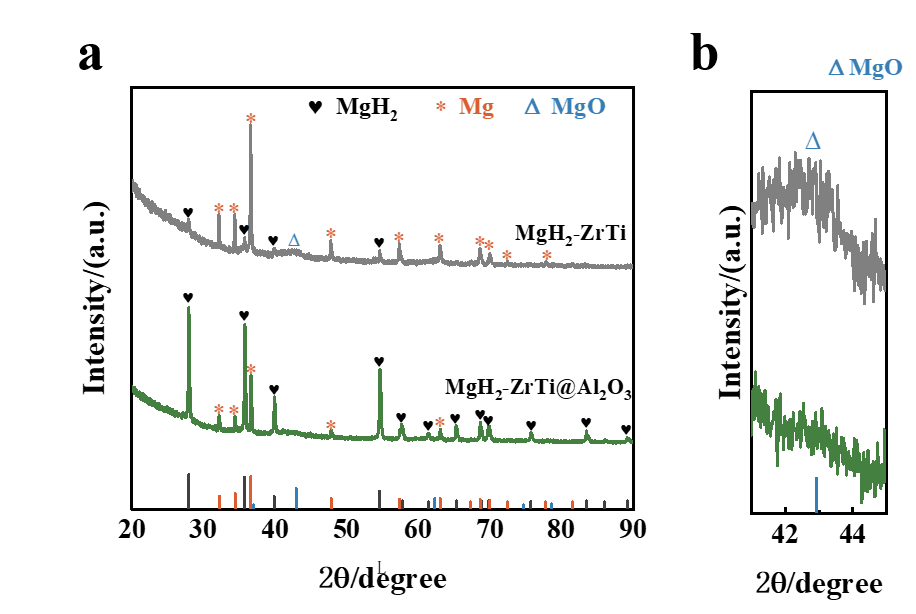


**Fig. S20** (**a**) XRD patterns and (**b**) partially enlarged XRD patterns of MgH_2_-ZrTi and MgH_2_-ZrTi@10nmAl_2_O_3_ after hydrogenation in 0.1%O_2_+0.4%N_2_+99.5%H_2_ atmosphere


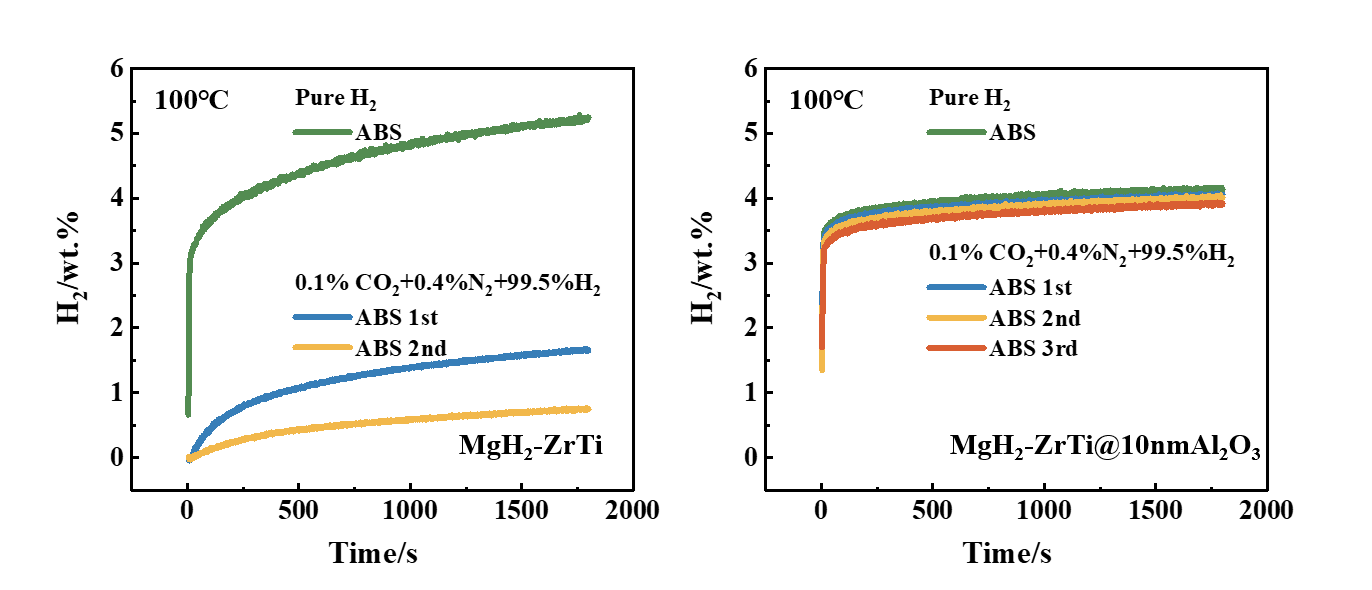


**Fig. S21** Isothermal hydrogenation curves in pure H_2_ and isothermal cyclic hydrogenation curves in 0.1%CO_2_+0.4%N_2_+99.5%H_2_ atmosphere (16 bar) for (**a**) MgH_2_-ZrTi and (**b**) MgH_2_-ZrTi@10nmAl_2_O_3_ at 100 ℃


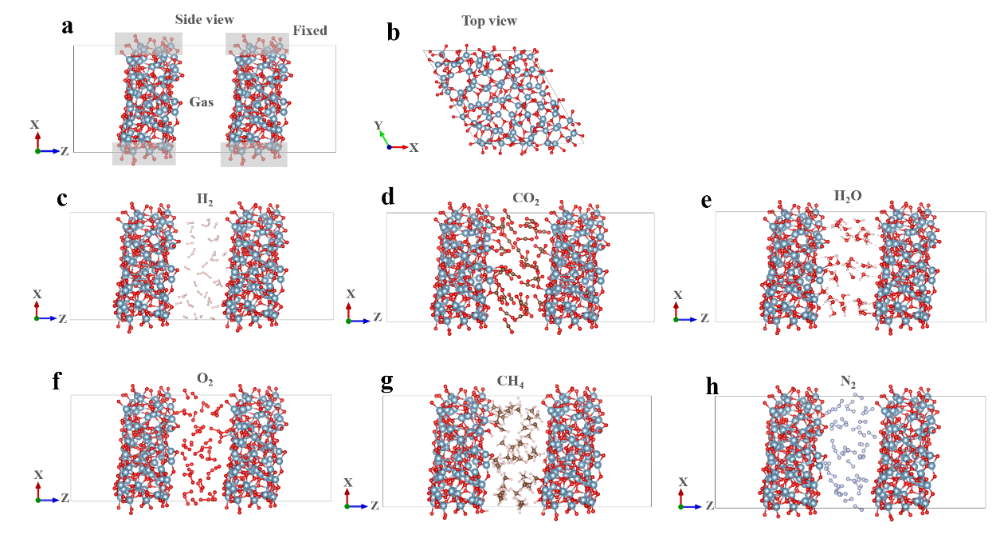


**Fig. S22 (a**) Side and (**b**) top views of the double-wall α- Al_2_O_3_ model consisting of two parallel slabs. (**c-h**) The simulation of the penetration of different gas molecule (H_2_, CO_2_, H_2_O, O_2_, CH_4_, and N_2_) between the two Al_2_O_3_ slabs


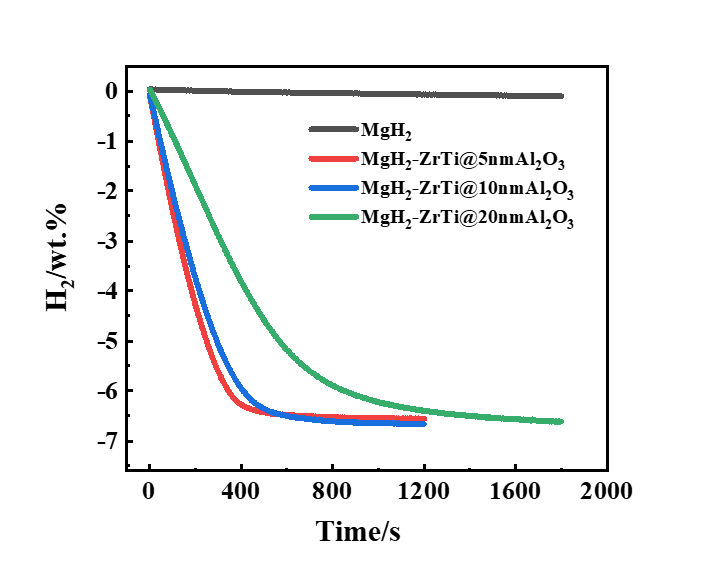


**Fig. S23** Normalized isothermal dehydrogenation (275 °C) curves of MgH_2_, MgH_2_-ZrTi@5nmAl_2_O_3_, MgH_2_-ZrTi@10nmAl_2_O_3_, and MgH_2_-ZrTi@20nmAl_2_O_3_ by MgH_2_ theoretical capacity


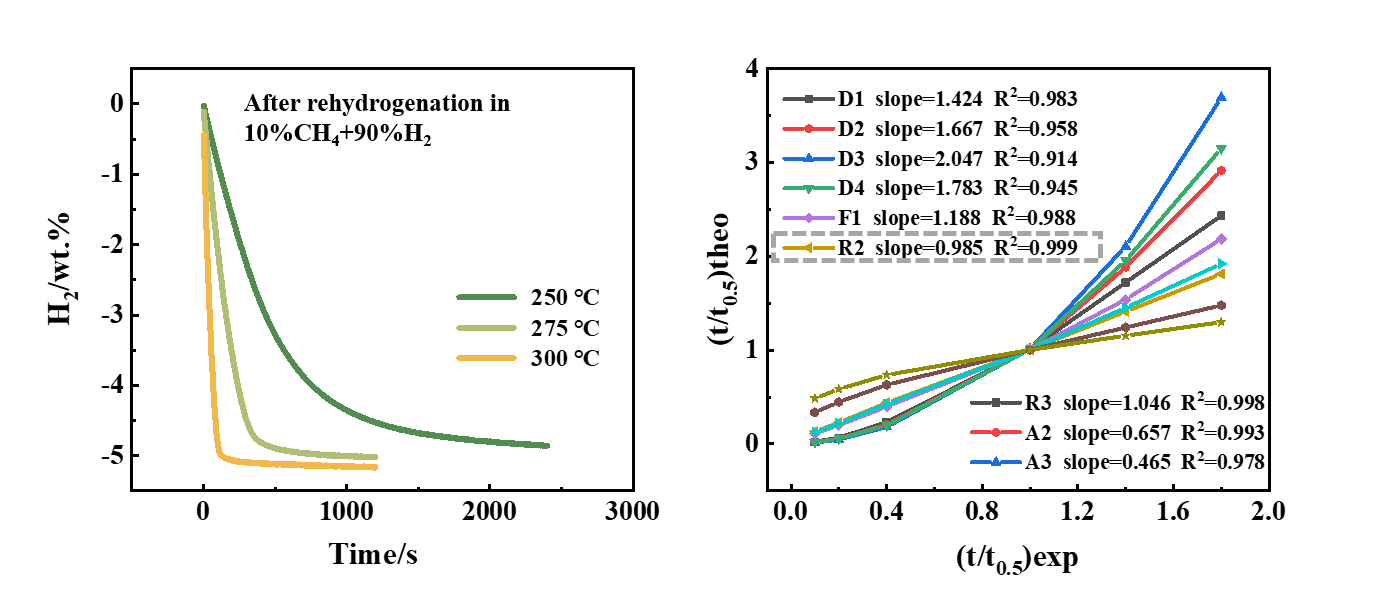


**Fig. S24** (**a**) Isothermal dehydrogenation curves of MgH_2_-ZrTi@10nmAl_2_O_3_ after re-hydrogenation at 100 ℃ for 1 h under 30 bar 10%CH_4_+90%H_2_ at different temperature and **(b**) the relationships of (t/t_0.5_)_theo_ vs. (t/t_0.5_)_exp_ at 275 ℃ according to various kinetic models


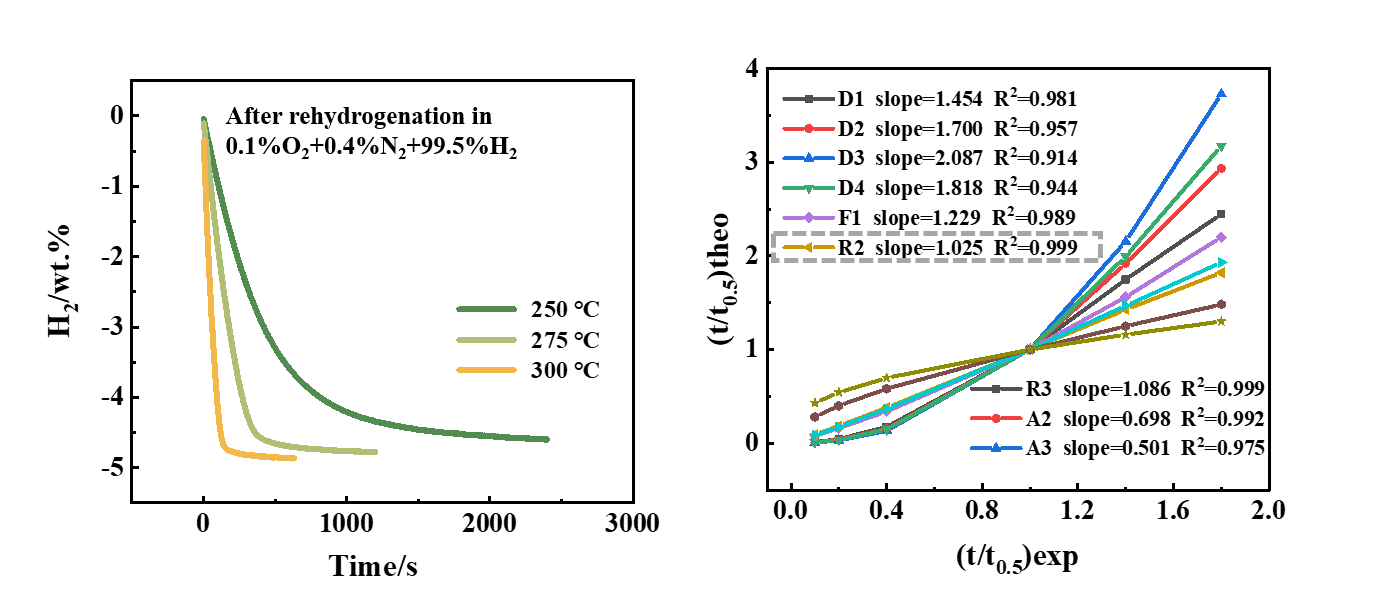


**Fig. S25** (**a**) Isothermal dehydrogenation curves of MgH_2_-ZrTi@10nmAl_2_O_3_ after re-hydrogenation at 100 ℃ for 1 h under 16 bar 0.1%O_2_+0.4%N_2_+99.5%H_2_ at different temperature and **(b**) the relationships of (t/t_0.5_)_theo_ vs. (t/t_0.5_)_exp_ at 275 ℃ according to various kinetic models


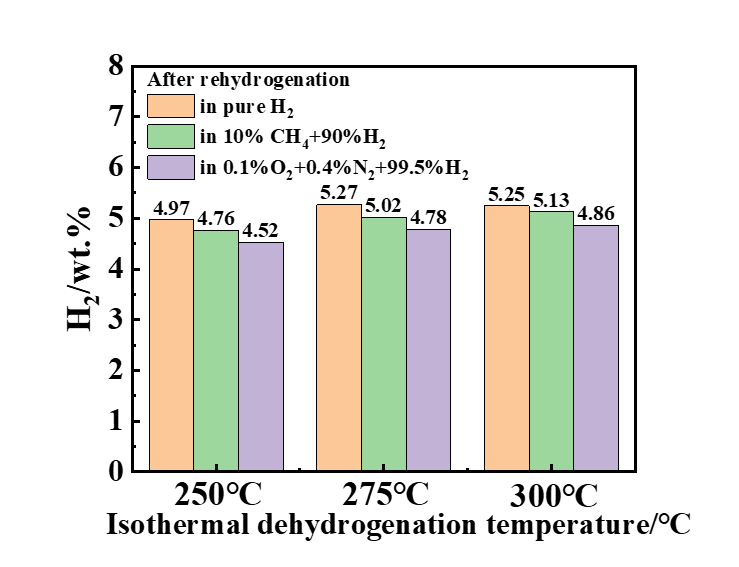


**Fig. S26** Dehydrogenation capacity of MgH_2_-ZrTi@10nmAl_2_O_3_ at 250 °C (within 30 min), 275 °C (within 20 min), and 300 °C (within 10 min) after re-hydrogenation in different hydrogen atmospheres


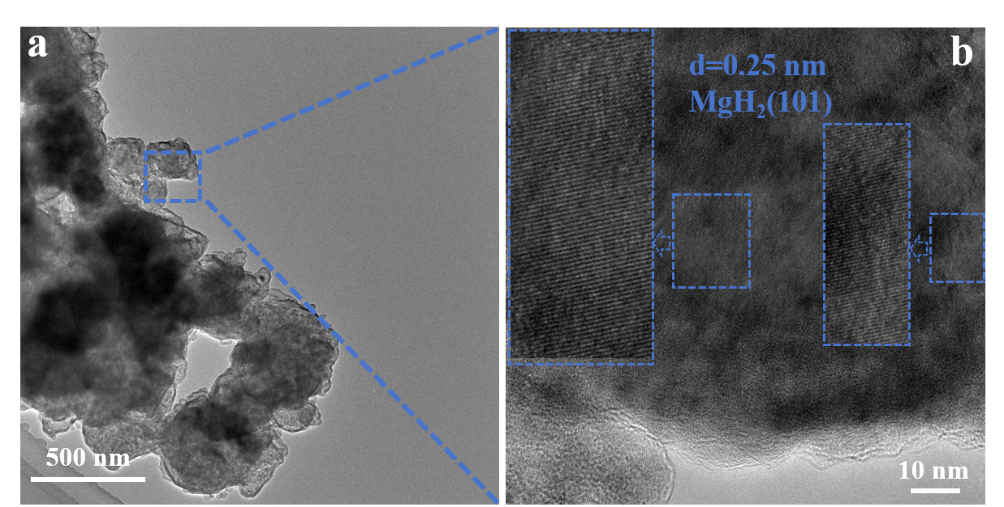


**Fig. S27** (**a**) TEM and (**b**) HRTEM of MgH_2_-ZrTi@10nmAl_2_O_3_ after rehydro-genation


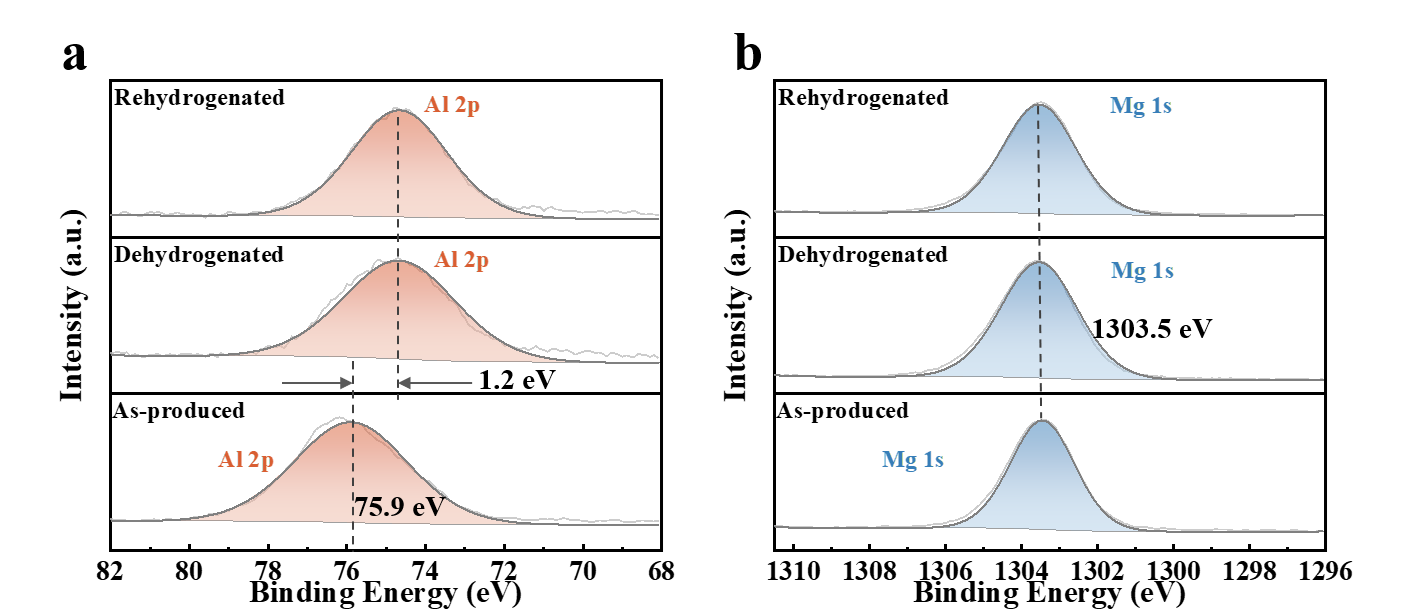


**Fig. S28** High-resolution XPS spectra of (**a**) Al 2p and **(b**) Mg 1s from the as-produced, dehydrogenated, and re-hydrogenated MgH_2_-ZrTi@10nmAl_2_O_3_


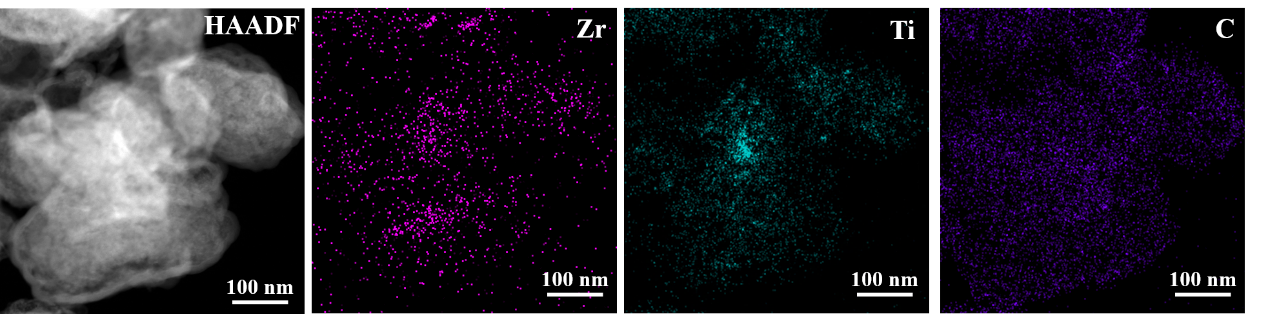


**Fig. S29** HAADF-STEM and the corresponding elemental analyses of MgH_2_-ZrTi@10nmAl_2_O_3_ after dehydrogenation


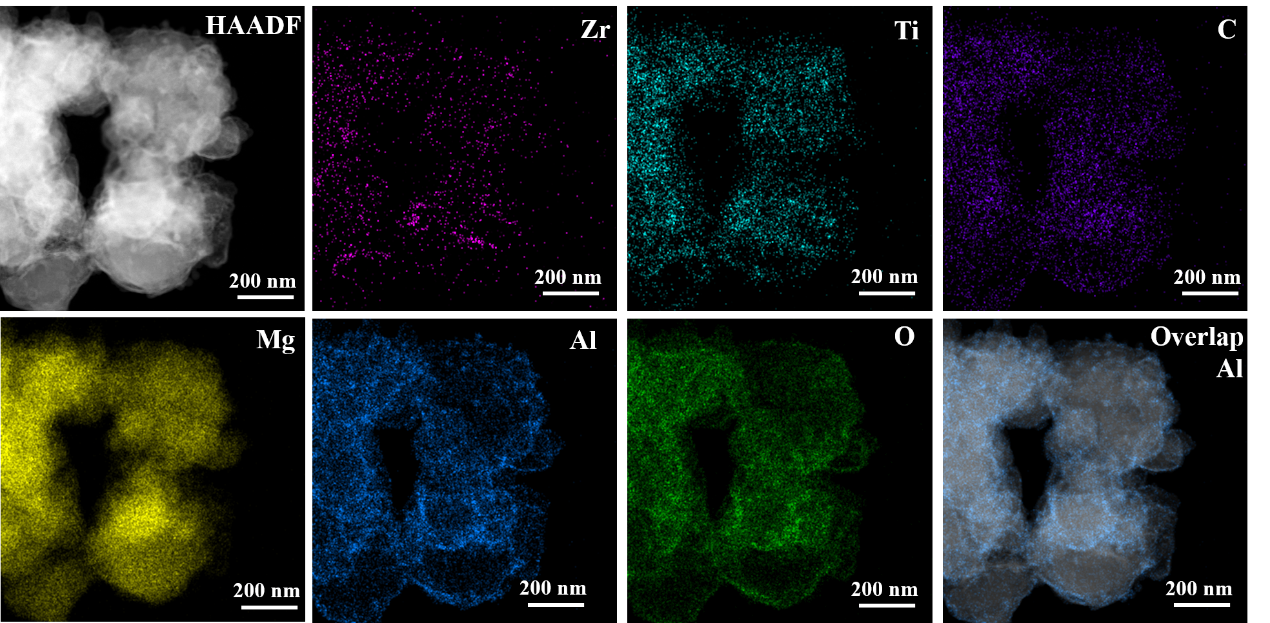


**Fig. S30** HAADF-STEM and the corresponding elemental analyses of MgH_2_-ZrTi@10nmAl_2_O_3_ after rehydrogenation


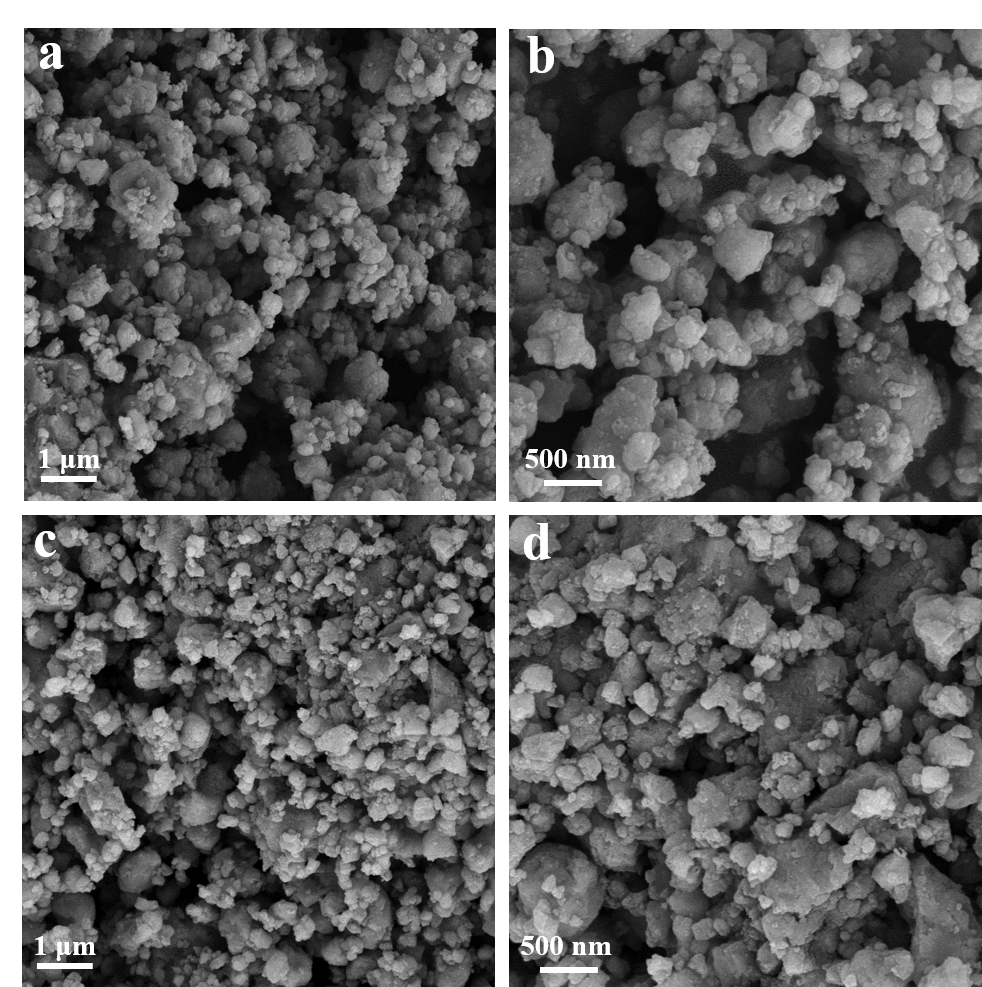


**Fig. S31** SEM images of MgH_2_-ZrTi@10nmAl_2_O_3_ after dehydrogenation at different scales


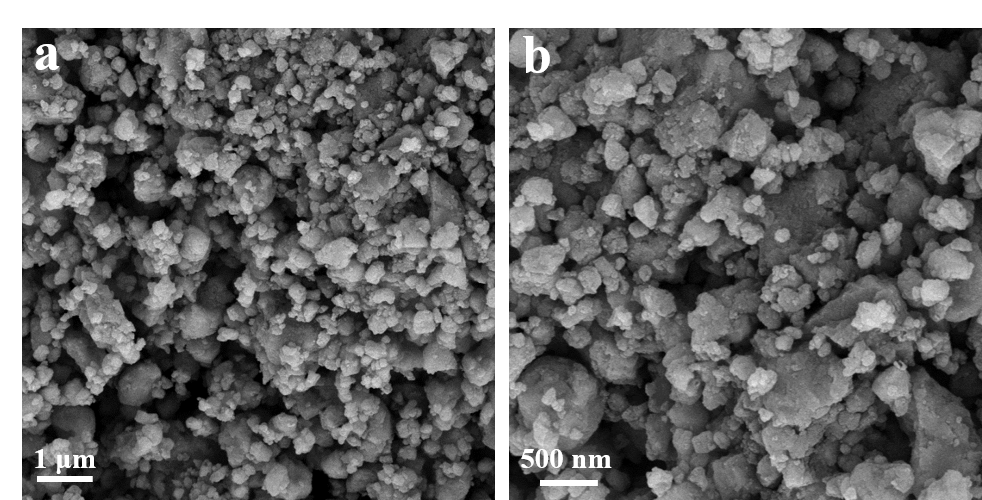


**Fig. S32** SEM images of MgH_2_-ZrTi@10nmAl_2_O_3_ after rehydrogenation at different scales

**Supplementary References**

1. C. Wu, Y. Wang, Y. Liu, W. Ding, C. Sun, Enhancement of hydrogen storage properties by *in situ* formed LaH3 and Mg_2_NiH_4_ during milling MgH_2_ with porous LaNiO3. Catal. Today **318**, 113–118 (2018). <https://doi.org/10.1016/j.cattod.2017.09.037>
2. A.P. Thompson, H.M. Aktulga, R. Berger, D.S. Bolintineanu, W.M. Brown et al., LAMMPS - a flexible simulation tool for particle-based materials modeling at the atomic, meso, and continuum scales. Comput. Phys. Commun. **271**, 108171 (2022). <https://doi.org/10.1016/j.cpc.2021.108171>
3. F. Bu, A. Wajid, M. Gu, T. Liu, S. Liu et al., Synergistic effect of multivalent Ti, Zr, and oxygen vacancies to significantly enhance the hydrogen sorption properties of MgH_2_. J. Mater. Chem. A **13**(21), 16102–16111 (2025). <https://doi.org/10.1039/D5TA01302J>
